# Seasonal and Organ-Specific Variations of Alkaloids in *Buxus obtusifolia* (Mildbr.) Hutch: A Multivariate LC/MS Study

**DOI:** 10.3390/plants15101439

**Published:** 2026-05-08

**Authors:** Justus Wambua Mukavi, Jandirk Sendker, Njogu M. Kimani, Leonidah Kerubo Omosa, Thomas J. Schmidt

**Affiliations:** 1 Pharma Campus, Institute of Pharmaceutical Biology and Phytochemistry (IPBP), University of Münster, Corrensstraße 48, D-48149 Münster, Germany; jmukavi@uni-muenster.de (J.W.M.); jandirk.sendker@uni-muenster.de (J.S.); 2 Department of Physical Sciences, University of Embu, P.O. Box 6, Embu 60100, Kenya; mark.njogu@gmail.com; 3 Department of Chemistry, Faculty of Science & Technology, University of Nairobi, P.O. Box 30197, Nairobi 00100, Kenya; lkerubo@uonbi.ac.ke

**Keywords:** *Buxus obtusifolia* (Mildbr.) Hutch, Buxaceae, aminosteroid alkaloids, seasonal variation, multivariate data analysis, principal component analysis, volcano plot, liquid chromatography–mass spectrometry

## Abstract

*Buxus obtusifolia* (Mildbr.) Hutch is an evergreen shrub endemic to East Africa and is traditionally used to treat chest ailments. Our recent investigation on the dichloromethane leaf extract of this species yielded several aminosteroid alkaloids, some of which demonstrated promising in vitro antiprotozoal activity warranting more detailed studies on this interesting plant and its bioactive constituents. Given that abiotic factors are known to influence the biosynthesis and accumulation of plant secondary metabolites, this study aimed to investigate seasonal and organ-specific variability in the alkaloid profile of *B. obtusifolia* to gain insights into the dynamics of their formation and, potentially, obtain hints at the best times to harvest individual alkaloids. Consequently, leaf and twig samples were collected each month from the same population over a period of one year and analyzed using ultra-high-performance liquid chromatography coupled with positive-mode electrospray ionization double quadrupole time-of-flight tandem mass spectrometry (UHPLC–ESI^+^–QqTOF–MS/MS). The resulting data, after conversion to <retention time: mass/charge ratio> (<*t*_R_:*m*/*z*>) variables, were analyzed by principal component analysis (PCA) to characterize variations in the metabolite profile. Evaluation of the first three principal components revealed clear differences between leaves and twigs, as well as subtle overall seasonal changes with some distinct dry-season clustering. A volcano plot was used to further analyze the differences between the minor constituents of the two organs. In total, 15 aminosteroid alkaloids were identified as key contributors to these differences. This represents the first seasonal and organ-specific phytochemical variability investigation in *B. obtusifolia*. Thus, this study offered the first valuable insights into the possible association of some abiotic factors and the phytochemical profile of this plant. Studies including further populations of this species from different locations will have to show whether the present findings allow general conclusions with respect to the investigated compounds’ accumulation in response to external factors. Furthermore, the present results represent a basis to delineate the optimal harvest period for targeted isolation of larger quantities of bioactive aminosteroids for further development.

## 1. Introduction

*Buxus obtusifolia* (Mildbr.) Hutch is an evergreen shrub endemic to Kenya and Tanzania, and holds a significant ethnomedicinal value among some East African communities who utilize its root decoctions to treat chest ailments [1]. Our recent phytochemical investigation of the species’ leaves led to the identification of 24 aminosteroid alkaloids [2]. Notably, some of these compounds exhibited promising in vitro antiprotozoal activity against *Trypanosoma brucei rhodesiense* (*Tbr*), the causative agent of East African Human Trypanosomiasis, and *Plasmodium falciparum* (*Pf*), the primary parasite responsible for tropical Malaria [2]. These findings prompted us to investigate the accumulation of the bioactive compounds in more detail. It is widely established that abiotic factors such as fluctuating temperatures, light intensity, nutrient availability, and water stress influence growth as well as biosynthesis and accumulation of active principles in medicinal plants [3]. Consequently, these environmental variations can significantly alter the overall therapeutic efficacy of the resulting herbal materials [4]. Numerous studies have demonstrated that the total alkaloid content in both higher plants [5,6,7] and their associated endophytic fungi [8,9] is greatly influenced by environmental factors such as the ones mentioned above. Using a multivariate data analysis (MVDA) approach, our group has recently evaluated seasonal, organ-specific and location variability in the alkaloid profiles of *Buxus sempervirens* var. *arborescens* and *suffruticosa* [10], and *Pachysandra terminalis* [11]. These investigations identified various aminosteroid alkaloids as the key contributors to the observed chemical differences among the plant organs (leaves, twigs and flowers) across seasons. Our recent study on *B. obtusifolia* represented the first phytochemical and pharmacological investigation on this species [2]. Accordingly, to the best of our knowledge, no previous studies have examined seasonal and organ-specific variability in its phytochemical profile. The aim of the present study was therefore to investigate the variations in the alkaloid content of *B. obtusifolia*, considering both the seasonal dynamics over a full annual vegetation cycle as well as differences between the primary aerial organs.

Samples of the leaves and twigs harvested monthly for one year were prepared and analyzed using ultra-high-performance liquid chromatography coupled with positive-mode electrospray ionization double quadrupole time-of-flight tandem mass spectrometry (UHPLC–ESI^+^–QqTOF–MS/MS, henceforth abbreviated LC/MS). In order to extract meaningful information from the resulting complex LC/MS data matrix, principal component analysis (PCA) and volcano plot were employed to identify the characteristic aminosteroid alkaloids responsible for seasonal variation and differences between leaves and twigs.

## 2. Results

### 2.1. Sample Preparation, LC/MS Characterization, and Data Preprocessing

To establish a seasonal profile, the leaves and twigs of *B. obtusifolia* were collected monthly over a period of one year (February 2024–January 2025). The air-dried plant materials were separately extracted with dichloromethane to obtain 24 extracts, which were subsequently analyzed using LC/MS. The resulting LC/MS raw data were processed using MetaboScape 3.0, where molecular features were extracted to generate a bucket table of 2195 <retention time:mass/charge ratio> (<*t*_R_:*m*/*z*>) variables from 84 MS chromatograms (24 × 3 measurements for leaf and twig samples and 12 measurements for a quality control sample). In order to identify seasonal trends, this complex dataset was imported to ProfileAnalysis 2.1 and evaluated using two multivariate statistical methods, principal component analysis (PCA) and volcano plot.

### 2.2. Quantification of Annual Variability of Previously Isolated B. obtusifolia Aminosteroid Alkaloids

**Figure 1:**
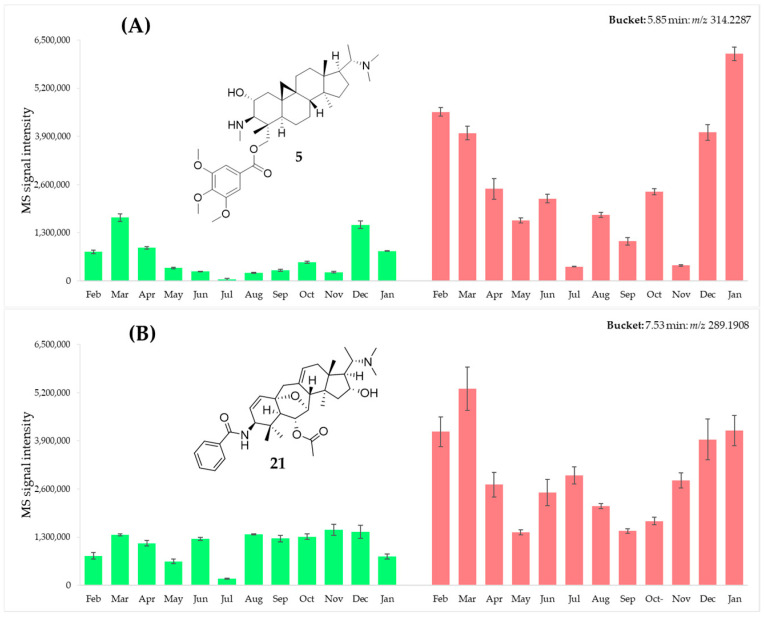
Monthly profiles of 29-trimethoxybenzoyloxy cycloprotobuxoline-C (**5**, (**A**)) (5.85 min: *m*/*z* 314.2287) and obtusiepoxamine-A (**21**, (**B**)) (7.53 min: *m*/*z* 289.1908). Bar charts are the mean signal intensities (±SD) of three replicate measurements across twelve monthly samples (February 2024–January 2025) of *B. obtusifolia* twigs (green) and leaves (red).

Our recent investigation reported 24 aminosteroids (**1**–**24**, Supplementary Materials, Figure S1) along with their antiprotozoal activity from the leaves of *B. obtusifolia* [2]. By comparing their exact retention times and mass-to-charge ratios <*t*_R_:*m*/*z*>, variables corresponding to each of the previously isolated bioactive compounds were traced within the bucket table, and their relative abundance across the 24 samples was assessed. In order to track the relative quantities of these constituents throughout an annual vegetative cycle, bar charts were constructed in Microsoft Excel to visualize the MS signal intensities across the sampling period. For each feature, the mean signal intensity of the three replicate measurements per month was calculated, with standard deviation represented as error bars. Samples were arranged chronologically from February 2024 to January 2025, allowing for a comparative analysis of each compound across the year. For instance, Figure 1 shows bar chart plots of 29-trimethoxybenzoyloxy cycloprotobuxoline-C (**5, **A) and obtusiepoxamine-A (**21**, B) with promising bioactivity [2], representing the 9*β*,10*β*-cyclo-5*α*-pregnane and 9 (10→19) *abeo*-5*α*-pregnane derivatives, respectively.

On average, both compounds are more abundant in the leaves (red bars) than in the twigs (green bars), showing the leaves as the preferred starting material for their isolation. Furthermore, 29-trimethoxybenzoyloxy cycloprotobuxoline-C (**5**), which exhibited the strongest antiplasmodial activity (IC_50_ = 0.5 μmol/L vs. 5.9 μmol/L for cytotoxicity; selectivity index, SI = 12) in our previous work [2], showed the highest concentration in January. In contrast, obtusiepoxamine-A (**21**), which showed moderate antitrypanosomal activity (IC_50_ = 2.2 μmol/L) and the highest selectivity index (SI = 16) among the 9 (10→19) *abeo*-5*α*-pregnane derivatives [2], reached its peak concentration in March. Additionally, both compounds **5** and **21** exhibited similar but minimally elevated concentrations, occurring in February–March for **5** and January–February for **21**, with both compounds also showing high levels in December. Overall, it can be deduced from these plots that the best time to harvest the leaves for isolating these two compounds would be from December to March.

Using the same criteria, the optimal harvest period for all *B. obtusifolia* aminosteroid alkaloids reported in our recent study [2] could be narrowed down (Supplementary Materials, Figure S2). The results (Table 1) indicated that, except for compounds **11**, **14** and **16**, all the other alkaloids exhibit their peak concentrations during the first quarter of the year. Additionally, except for compounds **8a** + **8b**, **11**, and **24**, the rest of the compounds were mainly found in higher quantities in the leaves compared to the twigs.

**Figure 2:**
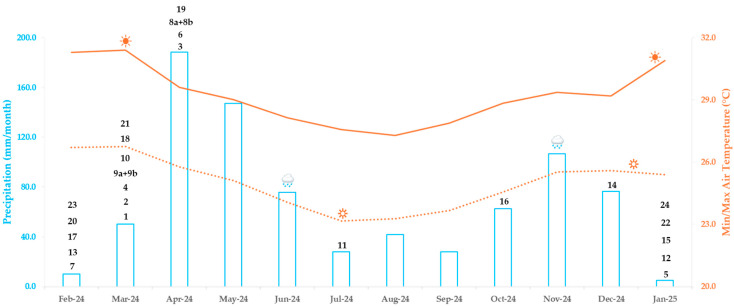
Months with maximum content of the previously isolated aminosteroid alkaloids (**1**–**24**) from the leaves of *B. obtusifolia* across a one-year cycle correlated with monthly weather data. Weather parameters from February to December 2024 are gridded data provided by the Kenya Meteorological Department, while those for January 2025 were estimated from a correlation plot between satellite-derived data and the gridded dataset (blue bars = monthly precipitation; orange solid line = monthly maximum temperature; and orange dotted line = monthly minimum temperature). Compound numbers correspond to those used in the text and in Table 1.

To correlate the optimum harvesting period with environmental conditions during the sampling period, we analyzed two abiotic factors, that is, temperature and precipitation. Abiotic stress, particularly drought and elevated temperatures, has been widely demonstrated to induce the accumulation of nitrogen-containing plant secondary metabolites, including alkaloids [12,13]. This is consistent with our observations, which showed accumulation of most compounds between January and March, coinciding with high temperatures (>30 °C) and low precipitation (Figure 2). Physiologically, drought and heat stress can trigger stress-response pathways, such as abscisic acid (ABA) signaling and oxidative stress mechanisms, which in turn upregulate the genes responsible for secondary metabolite biosynthesis [14,15]. Consequently, water-stressed plants often exhibit higher levels of defensive alkaloids that may contribute to stress tolerance or herbivore deterrence [16]. Furthermore, it is worth noting that reduced plant growth under water-limited conditions may lead to higher relative metabolite concentrations when expressed on a dry-weight basis.

### 2.3. Principal Component Analysis (PCA)

Principal component analysis (PCA) is a widely known unsupervised multivariate statistical method used to reduce the dimensionality of complex datasets while retaining the maximum possible variance [17]. PCA simplifies data interpretation with minimal loss of information by transforming a large set of correlated variables into a smaller number of orthogonal, uncorrelated variables known as principal components (PCs). The first principal component (PC1) captures the highest degree of variability, while subsequent components (PC2 and PC3, amongst others) explain the remaining variance in descending order [18]. Following the methodology established in previous studies by our group [10,11], PCA models were calculated and cross-validated using three different scaling approaches (level, unit variance, and Pareto scaling) as well as analysis of the unscaled data. When compared, all four approaches produced models that effectively discriminated between leaf and twig samples of *B. obtusifolia* (Supplementary Materials, Figure S3). The Pareto-scaled model was selected for further analysis, as it provides the most balanced representation of variance between high and low abundance metabolites, as previously described by both Szabó and Schmidt and Schäfer et al. of our group [10,11].

**Figure 3:**
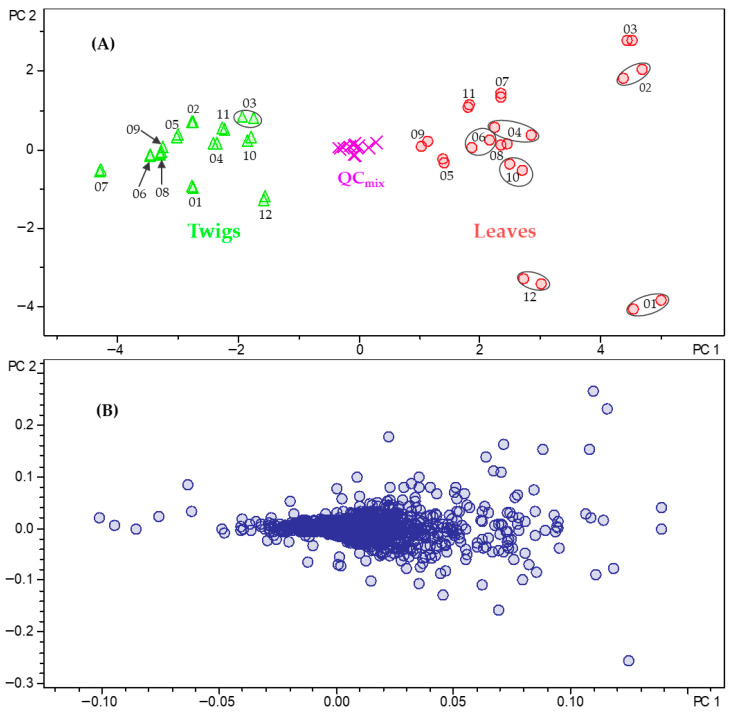
PCA model showing the scores (**A**) and loadings (**B**) plots based on Pareto-scaled data for the annual metabolite profile of *B. obtusifolia*. PC2 (10.0% of the total variance) is plotted against PC1 (43.2% of the total variance). In the scores plot (**A**), green triangles = twigs, red dots = leaves, and pink crosses = quality control (QC_mix_). Numerical values (01–12) indicate the months from January to December. Ellipses connect analyses of the same sample in cases where the symbols do not overlap.

Using ProfileAnalysis 2.1, samples are visualized in a score plot, which shows their relative positions in the PC space and enables the identification of natural groupings or trends. The corresponding loadings plot is used to determine which original variables contribute most significantly to each principal component. The results of the PCA model obtained with the Pareto-scaled data are presented in Figure 3.

The scores plot (Figure 3A), displaying the second PC (PC2) plotted against the first PC (PC1), clearly demonstrates a separation between the two organs of *B. obtusifolia*. PC1 differentiates between the leaf and twig samples and accounts for 43.2% of the total variance, while PC2 explains an additional 10.0% of the variance and reflects the temporal variability of the samples. Together, the scores plot of PC2 vs. PC1 captures 53.2% of the total variance in the bucket table. The quality control (QC_mix_), consisting of an equal mixture of all samples, is located in the middle of the plot as expected. The associated loadings plot (Figure 3B) shows the distribution of bucket variables <*t*_R_:*m*/*z*> within the PC coordinate system. Variables characteristic of the leaves are located on the right (high positive values on PC1), whereas those characteristic of twigs are on the left (strongly negative values on PC1). Consequently, these variables represent the primary contributors to the observed differences between the two plant organs. Interestingly, PC2 appears to reflect a relatively large difference between December and January (negative scores on PC2) and February and March (positive scores on PC2).

### 2.4. Identification of Aminosteroids Contributing to the Chemical Differences Between the Organs and Temporal Variation

**Figure 4:**
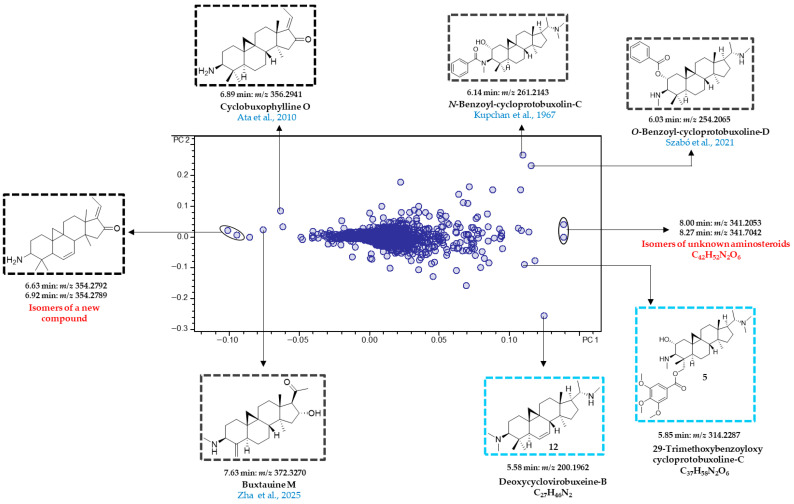
PCA loadings plot (PC2 vs. PC1) of the organ-specific metabolite profile of *Buxus obtusifolia*. Compounds highlighted in blue were previously isolated in our study [2], while those in black were tentatively identified based on their molecular masses using the SciFinder and LOTUS databases. The cited references in the figure are Kupchan et al., 1967 [22], Ata et al., 2010 [23], Szabó et al., 2021 [24], and Zha et al., 2025 [25].

The loadings plot (Figure 4) was examined to extract the molecular masses of the most influential bucket variables, to identify the aminosteroids responsible for differences between leaf and twig samples. The molecular formulae of these variables were determined by analyzing the corresponding +ESI-QqTOF MS/MS spectra and compared with the literature data as well as previously isolated compounds from *B. obtusifolia* [2]. Furthermore, the fragmentation patterns of these variables were evaluated and compared with diagnostic fragments characteristic of the steroidal skeletons found in *Sarcococca* [19], *Pachysandra* [20], and *Buxus* [21], all members of the Buxaceae family. These diagnostic fragments have been previously compiled and described in earlier studies of our group [10,11].

Among the leaf-specific compounds, 29-trimethoxybenzoyloxy cycloprotobuxoline-C (**5**) (5.85 min:*m*/*z* 314.2287) and deoxycyclovirobuxeine-B (**12**) (5.58 min:*m*/*z* 200.1962) were identified as characteristic constituents of the leaves of *B. obtusifolia*. Notably, these two compounds demonstrated the highest antiplasmodial (IC_50_ = 0.5 μmol/L for compound **5**) and antitrypanosomal activity (IC_50_ = 0.8 μmol/L for compound **12**) in our recent study on the leaves of this species [2]. Correlation with the scores plot (Figure 3A) further indicates that deoxycyclovirobuxeine-B (**12**) is most strongly associated with leaf samples collected in January and December. Similarly, *O*-benzoyl-cycloprotobuxoline-D (6.03 min:*m*/*z* 254.2065) and *N*-benzoyl-cycloprotobuxolin-C (6.14 min:*m*/*z* 261.2143) were also identified as characteristic compounds of the leaves. The former was previously reported from *Buxus sempervirens* L. by Szabó et al. [24], while the latter was described from the same species by Kupchan et al. [22]. Based on the positions of the corresponding buckets in the loadings plot (Figure 4), the two compounds are characteristic of samples collected in February and March (Figure 3A). Cyclobuxophylline O (6.89 min:*m*/*z* 356.2941) and buxtauine M (7.63 min:*m*/*z* 372.3270), as well as a pair of previously undescribed isomeric aminosteroids (6.63 min:*m*/*z* 354.2792 and 6.92 min:*m*/*z* 354.2789), were identified as characteristic compounds of the twigs of *B. obtusifolia*. Finally, all the 24 compounds described in our previous study from this species could be located in the PC2 vs. PC1 loadings plot (Supplementary Materials, Figure S4). Among these, compounds **1**, **2**, **4**, **5**, **10**, **12**, **16**, **21**, and **22** were found to significantly influence the organ-specific PCA model.

### 2.5. Seasonal Differences in the Annual Alkaloid Profile of Buxus obtusifolia

**Figure 5:**
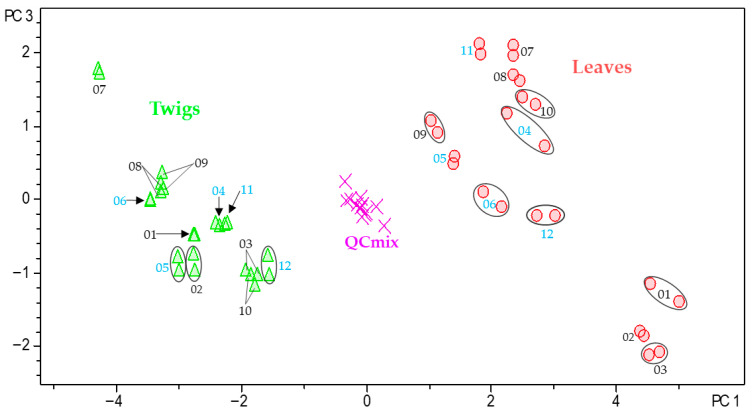
PCA scores plot (PC3 vs. PC1) of the seasonal metabolite profile of *Buxus obtusifolia*. Numerical values (01–12) indicate the months from January to December. Months highlighted in blue and black correspond to the rainy and dry seasons, respectively. Symbols and colors represent the same as in Figure 3A. Ellipses connect analyses of the same sample in cases where the symbols do not overlap.

To further investigate the seasonal variability in the alkaloid profile of *B. obtusifolia*, the third principal component (PC3) was plotted against the first (PC1) (Figure 5). Since PC3 accounts for an additional 7.0% of the variance, the scores plot of PC3 vs. PC1 captures a total of 50.2% of the overall variance (i.e., PC1–PC3 altogether capture 60.2%).

Based on monthly precipitation data provided by the Kenya Meteorological Department (Figure 2), samples were classified into rainy and dry seasons. Months with an average precipitation > 70 mm (April, May, June, November and December) were designated as the “rainy season,” while the remaining months were considered as the “dry season.” Given that sampling was conducted in a tropical region, temperature variation across the study period was minimal, except in January–March, when average monthly temperatures exceeded 30 °C (Figure 2). This relatively stable temperature pattern likely explains the absence of a clear and consistent seasonal separation or obvious sequential variation among the samples. However, subtle trends indicating an association with seasonal changes could be observed. For example, the leaf samples collected during the warmest months (January-March) cluster in the lower right region of the scores plot (Figure 5), indicating that the corresponding bucket variables represent compounds characteristic of the dry and warmest season. In contrast, the twig samples collected in July appear distinctly separated in the upper left region of the scores plot.

**Figure 6:**
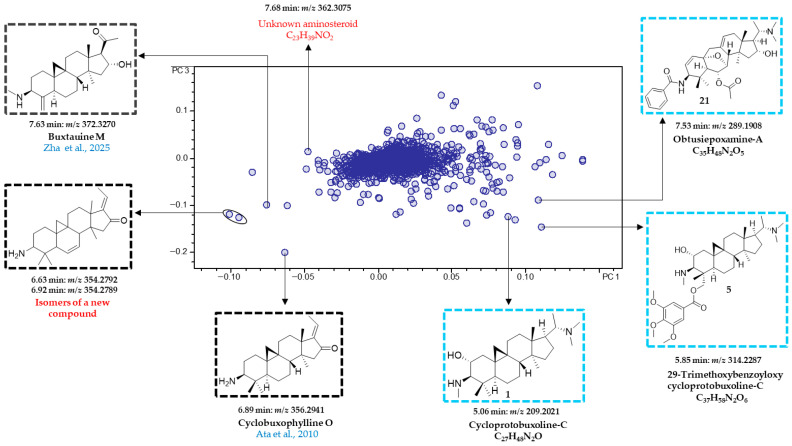
PCA loadings plot (PC3 vs. PC1) of the seasonal metabolite profile of *Buxus obtusifolia*. Compounds highlighted in blue were previously isolated in our study [2], while those in black were tentatively identified based on their molecular masses using the SciFinder and LOTUS databases. The cited references in the figure are Ata et al., 2010 [23], and Zha et al., 2025 [25].

Accordingly, cycloprotobuxoline-C (**1**) (5.06 min:*m*/*z* 209.2021), 29-trimethoxybenzoyloxy cycloprotobuxoline-C (**5**) (5.85 min:*m*/*z* 314.2287), and obtusiepoxamine-A (**21**) (7.53 min:*m*/*z* 289.1908) (Figure 6) previously isolated in our study [2] were identified as characteristic of leaf samples from the warmest season. Apart from an aminosteroid with the molecular formula C_23_H_39_NO_2_ (7.68 min:*m*/*z* 362.3075) previously undescribed for *B. obtusifolia* and Buxaceae, no other buckets were associated with twig samples collected during the dry and coolest month of July. In contrast, cyclobuxophylline O (6.89 min:*m*/*z* 356.2941), buxtauine M (7.63 min:*m*/*z* 372.3270), and the two new isomers (6.63 min:*m*/*z* 354.2792 and 6.92 min:*m*/*z* 354.2789) are likely characteristic of the twig samples collected during the period from October to May located in this region of the PC3 vs. PC1 scores plot (see Figure 5).

### 2.6. Volcano Plot-Based Comparison of Leaves and Twigs Alkaloid Profile

**Figure 7:**
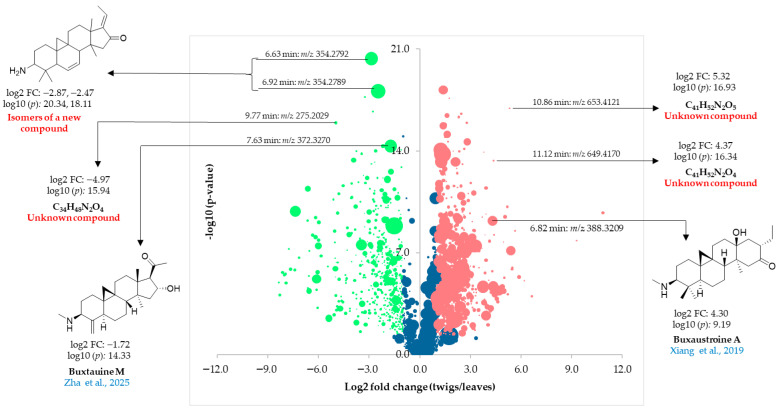
Volcano plot of twig and leaf annual samples from *B. obtusifolia*. Data points represent molecular features defined by their <*t*_R_:*m/z*> plotted as the mean log2 fold change against −log10(*p*-value), and bubble sizes are proportional to the maximum signal intensity observed across all 24 samples. Significant features in twig and leaf samples are highlighted in green and red, respectively, while non-significant features are shown in blue. Significance thresholds were set at|log2 fold change| ≥ 1 and *p*-value < 0.05. The cited references in the figure are Zha et al., 2025 [25] and Xiang et al., 2019 [26].

The two PCA models (PC2 vs. PC1 and PC3 vs. PC1) revealed a clear separation between the leaf and twig samples of *B. obtusifolia* and highlighted the major compounds contributing to this separation. However, compounds present in low concentrations are normally overlooked due to their limited influence on PCA models and are therefore difficult to identify through this approach alone. Such constituents, despite their low abundance, may be of interest since they could, e.g., represent biosynthetic intermediates, degradation products, or novel compounds with potential pharmacological relevance. To complement the PCA results, a volcano plot based on direct pairwise *t*-test comparison between the two organs was therefore performed. Each bucket was individually evaluated by plotting the statistical significance of the difference between the leaves and twigs (−log10(*p*-value)) against the magnitude of change (log2(fold change). This allowed visualization of both the magnitude and significance of concentration differences without less abundant compounds being dominated by the more abundant ones. From Figure 7, bucket variables corresponding to leaf compounds occur to the far right on the *x*-axis, while those occurring mainly in the twigs are on the left side of the *x*-axis. Out of the 2195 variables, 1190 showed statistically significant differences between leaves and twigs (*p*-value < 0.05, |log2 fold change| ≥ 1), with 510 features concentrated in the twigs (green bubbles) and 680 concentrated in the leaves (red bubbles). Notably, all variables with the highest PCA loadings (PC2 vs. PC1, Figure 4) were also statistically significant in the volcano plot, although they were not necessarily among the most differentially expressed variables. This finding is consistent with observations reported for *B. sempervirens* in the earlier study by our group [10].

Among the significant compounds, buxtauine M (7.63 min:*m/z* 372.3270) and the two new isomers (6.63 min:*m/z* 354.2792 and 6.92 min:*m/z* 354.2789), all of which had been identified as characteristic compounds of the twigs of *B. obtusifolia* (Figure 4), were additionally detected to be significantly more concentrated in the twigs than in the leaves (Figure 7). The volcano plot also highlighted another minor variable belonging to a previously unknown compound (9.77 min:*m/z* 275.2029) with a high log2(fold-change) of −4.97 as a characteristic compound of the twigs. Conversely, buxaustroine A (6.82 min:*m/z* 388.3209) was shown to be in significantly higher concentration in the leaves compared to the twigs. Two additional variables (10.86 min:*m/z* 653.4121 and 11.12 min:*m/z* 649.4170) exhibited particularly substantial log2(fold-changes) of 5.32 and 4.37, respectively. Based on their molecular mass, these variables were attributed to minor, previously unreported aminosteroids that are concentrated in the leaves.

## 3. Materials and Methods

### 3.1. Plant Material Processing and Extraction

The aerial parts of *Buxus obtusifolia* were harvested monthly from the same population site described in our previous study [2] over a period of one year (February 2024–January 2025). Identification of the plant material was done by Mr. Patrick Mutiso, a taxonomist from the Faculty of Science and Technology, University of Nairobi. The voucher specimens were deposited at both the University of Nairobi Herbarium (UoN_JM 2022_002) and at the Institute of Pharmaceutical Biology and Phytochemistry, University of Münster (IPBP 916-TS_JM_2022_002). To complement the seasonal alkaloid variation, organ-specific variability was also evaluated by separately processing the leaves and twigs. The plant materials were air-dried under shade at room temperature to a constant weight and pulverized into fine powder using a mill. For each sample, 5 g of the powdered material was exhaustively extracted with dichloromethane (2 × 30 mL; same solvent as used in our previous study [2]) under continuous agitation on a magnetic stirrer (IKA-Werke GmbH & CO. KG, Staufen, Germany) for 30 min. The obtained extracts were combined and thoroughly evaporated under reduced pressure at 40 °C.

### 3.2. UHPLC/+ESI-QqTOF-MS/MS-Analysis

The resulting extracts were analyzed using the previously described LC/MS method by our research group [11], with minor changes. The extracts were dissolved in methanol at a concentration of 10 mg/mL and analyzed using a Bruker Daltonics micrOTOFQII time-of-flight mass spectrometer (Bruker Daltonics GmbH, Bremen, Germany). Chromatographic separation was achieved using a binary gradient of H_2_O (+0.1% formic acid; A) and Acetonitrile (+0.1% formic acid; B) at a flow rate of 0.4 mL/min and column temperature of 40 °C. The elution profile described in our recent article [2] was used: 0–1.88 min: linear from 15% B to 30% B; 1.88–7.88 min: linear from 30% B to 33% B; 7.88–9.9 min: linear from 33% B to 50% B; 9.9–9.93 min: linear from 50% B to 100% B; 9.93–15.88: isocratic 100% B; 15.88–15.98 min: linear from 100% B to 15% B; 15.98–20.0 min: isocratic 15% B. In order to monitor injection precision and instrument sensitivity, 10 µL of papaverine (0.25 mg/mL) was added to each sample as an internal standard. For quality control, 20 μL of each sample was pooled into a collective LC/MS vial (QCmix) and measured after every six samples to assess sensitivity shifts and peak alignment consistency. Additionally, a methanol blank was measured after every 20 samples to monitor any carry-over of analytes. To obtain more information about the fragmentation patterns, some representative samples were initially subjected to auto MS/MS analysis at a collision energy of 20 eV. These initial runs also served to saturate the column matrix, ensuring reproducible retention times throughout the sequence. Following the matrix saturation measurements, the complete sample set was analyzed in triplicate using full-scan mode without intermediate recording of fragment spectra to obtain the maximum number of data points per peak. An injection volume of 2 µL was employed for all analyses. The robustness of the dataset was ensured by measuring the samples in three distinct sequences: once sequentially from January 2025 to December 2024, followed by a randomized sequence and concluding with a sequential repetition.

### 3.3. Preprocessing of LC/MS Data

The raw LC/MS data from 84 sample measurements (24 × 3 measurements for leaf and twig samples and 12 measurements for a quality control sample) were processed using MetaboScape 3.0 software (Bruker Daltonics, Bremen, Germany). Molecular features were extracted to generate a comprehensive bucket table of <*t*_R_:*m*/*z*> variables. The sample groups were defined by organ (leaves and twigs) and harvest month (January–December). The filter parameters were chosen in accordance with the values used successfully in our previous studies [10,11]. They were defined as follows: minimum features for extraction, 20/84 (accounting for molecular features detected in at least 20/84 samples during extraction); presence of features, 20/84 (representing features only present in at least 20/84); and filter features by occurrence in groups, month 10% (accounting for features occurring in at least 10% of the samples within each group; in this case, the groups represented the different organs, leaves/twigs). The peak detection parameters were set as: intensity threshold, 1000 counts; minimum peak length, 8 spectra; minimum peak length (recursive), 7 spectra; retention time range, 1–15 min; and mass range, *m*/*z* 50–1500. Ion deconvolution parameters were set as: EIC correlation, 0.7; primary ions, [M + H]^+^; seed ions, [M + 2H]^2+^, [M + Na]^+^, and [M + K]^+^; and common ions, [M + H−H_2_O]^+^. The resulting bucket table containing 2195 variables x 84 analyses was exported to Bruker ProfileAnalysis 2.1 software (Bruker Daltonics, Bremen, Germany) for multivariate statistical evaluation.

### 3.4. Principal Component Analysis (PCA) Modeling

Prior to PCA modeling, the 2195 bucket variables’ intensity values of each analysis were normalized to those of the internal standard papaverine (*t*_R_ 5.84 min:*m*/*z* 340.1580) in the respective run. To optimize the statistical output, three different scaling approaches were evaluated, such as level, unit variance, and Pareto, as well as analysis of the unscaled data (Supplementary Materials, Figure S3). All PCA models were cross-validated using leave-one-out cross-validation to ensure robustness of the resulting data. While all four approaches demonstrated good sample separation, the Pareto-scaled model was selected for further analysis due to its superior representation of the variance between high and low abundance metabolites.

### 3.5. Volcano Plot

Using the bucket table exported from MetaboScape, a *t*-test was performed in Microsoft Excel to compare mean signal intensities between twig and leaf samples of *B. obtusifolia*. Log2 fold change and −log10(*p*-value) were calculated for each bucket and visualized as a bubble volcano plot, with bubble size proportional to the maximum signal intensity observed across all samples. Significance thresholds were set at |log2 fold change| ≥ 1 and *p*-value < 0.05.

## 4. Conclusions

The present study investigated variations in the alkaloid content of the leaf and twig samples of *B. obtusifolia* over a full annual vegetation cycle using a multivariate data analysis approach. Direct comparison of the intensity of mass signals over the months for each of the bioactive aminosteroids identified in our previous study helped to narrow down the probably best time periods for their targeted isolation. Principal component analysis and volcano plot of the LC/MS dataset revealed clear organ-specific differences in alkaloid profiles, in addition to some less clear-cut trends in seasonal metabolite variation. In total, 15 compounds were identified as key contributors to the observed patterns. Additionally, among the 24 aminosteroids described in our previous study from this species, compounds **1**, **2**, **4**, **5**, **10**, **12**, **16**, **21**, and **22** exhibited the most profound organ-specific differences as reflected in the PCA model. Future studies with different populations of *B. obtusifolia* will have to show whether the present findings on the investigated compounds’ accumulation in response to external factors can be generalized. Since no controlled environmental experiments could be conducted in the present study, rendering conclusions regarding the effects of abiotic stress on the alkaloid profile inferential, future experiments under controlled greenhouse conditions would certainly be desirable. The present findings provide valuable first insights into the phytochemical dynamics of the investigated population of *B. obtusifolia* and offer a basis for the targeted isolation of bioactive aminosteroids in future pharmacological studies.

## Figures and Tables

**Table 1:** Month of maximum content and preferred starting material for isolation of previously reported bioactive aminosteroids from *B. obtusifolia*. The compounds are characterized by their retention times (minutes) and the mass/charge ratios (*m*/*z*) of their diagnostic mass signals. They are numbered according to our previous publication [2].

Compound	Name	Bucket <*t*_R_:*m*/*z*>		Type of Ion	Elemental Formula of Ion	Month with Maximum Content	Organ
		***t*** **_R_ (min)**	***m*** **/** ***z***				
**1**	Cycloprotobuxoline-C	5.06	209.2021	[M + 2H]^2+^	C_27_H_50_N_2_O^2+^	March	Leaves > twigs
**2**	Cycloprotobuxoline-C *N*_20_-oxide	4.63	217.1995	[M + 2H]^2+^	C_27_H_50_N_2_O_2_^2+^	March	Leaves > twigs
**3**	16α-Hydroxycycloprotobuxoline-C	4.74	217.1996	[M + 2H]^2+^	C_27_H_50_N_2_O_2_^2+^	April	Leaves > twigs
**4**	Cycloprotobuxoline-D	5.05	202.1941	[M + 2H]^2+^	C_26_H_48_N_2_O^2+^	March	Leaves > twigs
**5**	29-Trimethoxybenzoyloxy cycloprotobuxoline-C	5.85	314.2287	[M + 2H]^2+^	C_37_H_60_N_2_O_6_^2+^	January	Leaves > twigs
**6**	*N*_3_-Demethylcycloprotobuxoline-C	4.84	403.3730	[M + H]^+^	C_26_H_47_N_2_O^+^	April	Leaves > twigs
**7**	16α-Hydroxy-*N*_3_-demethylcycloprotobuxoline-C	5.66	419.3675	[M + H]^+^	C_26_H_47_N_2_O_2_^+^	February	Leaves > twigs
**8a + 8b**	Cycloprotobuxoline-D *N*_3_-*trans*- (**8a**) and cycloprotobuxoline-D *N*_3_-*cis* (**8b**) -formamide	6.46	431.3647	[M + H]^+^	C_27_H_47_N_2_O_2_^+^	April	Leaves ≈ twigs
**9a + 9b**	16α-Hydroxycycloprotobuxoline-C *N*_3_-*trans*-formamide (**9a**) and 16α-hydroxycycloprotobuxoline- C *N*_3_-*cis*-formamide (**9b**)	7.76	461.3553	[M + H]^+^	C_28_H_49_N_2_O_3_^+^	March	Leaves > twigs
**10**	Cyclonataminol	4.89	223.1997	[M + 2H]^2+^	C_28_H_50_N_2_O_2_^2+^	March	Leaves > twigs
**11**	*N*_3_-Demethyl cyclonataminol	4.70	216.1917	[M + 2H]^2+^	C_27_H_48_N_2_O_2_^2+^	June	Leaves ≈ twigs
**12**	Deoxycyclovirobuxeine-B	5.58	200.1962	[M + 2H]^2+^	C_27_H_48_N_2_O^2+^	January	Leaves > twigs
**13**	Cyclovirobuxeine-A	5.25	215.2018	[M + 2H]^2+^	C_28_H_50_N_2_O^2+^	February	Leaves > twigs
**14**	Cyclovirobuxeine-B	5.29	208.1937	[M + 2H]^2+^	C_27_H_48_N_2_O^2+^	December	Leaves > twigs
**15**	*N*_20_-Demethyl deoxycyclobuxoxazine A	5.42	208.2079	[M + 2H]^2+^	C_27_H_48_N_2_O^2+^	January	Leaves > twigs
**16**	Obtusibuxeine A	6.72	374.3053	[M + H]^+^	C_24_H_40_NO_2_^+^	October	Leaves >> twigs
**17**	*O*^10^-Obtusifuranamine-A	6.30	593.3572	[M + H]^+^	C_35_H_49_N_2_O_6_^+^	February	Leaves > twigs
**18**	*O*^10^-Obtusifuranamine-B	7.03	655.3697	[M + H]^+^	C_40_H_51_N_2_O_6_^+^	March	Leaves > twigs
**19**	16-Deoxy-*O*^10^-obtusifuranamine-B	7.55	639.3775	[M + H]^+^	C_40_H_51_N_2_O_5_^+^	April	Leaves > twigs
**20**	*O*^2^-Natafuranamine	6.74	593.3559	[M + H]^+^	C_35_H_49_N_2_O_6_^+^	January	Leaves > twigs
**21**	Obtusiepoxamine-A	7.53	289.1908	[M + 2H]^2+^	C_35_H_50_N_2_O_5_^2+^	March	Leaves > twigs
**22**	Obtusidienolamine-A	7.04	579.3763	[M + H]^+^	C_35_H_51_N_2_O_5_^+^	January	Leaves > twigs
**23**	Deoxyobtusidienolamine-A	7.23	563.3813	[M + H]^+^	C_35_H_51_N_2_O_4_^+^	February	Leaves > twigs
**24**	Obtusiaminocyclin	5.92	368.2594	[M + H]^+^	C_24_H_34_NO_2_^+^	January	Leaves ≈ twigs

## Data Availability

All original data for this study are detailed in the article and Supplementary Materials. The raw data supporting the findings of this study are available from the authors upon request.

## Supplementary Materials

The following supporting information can be downloaded at: https://www.mdpi.com/article/10.3390/plants15101439/s1, Figure S1: Chemical structures of aminosteroids previously isolated from *Buxus obtusifolia* in our recent study [2]; Figure S2: Monthly profiles of compounds **1**–**4**, **6**–**20**, and **22**–**24**; Figure S3: PCA models of the LC/MS data of *B. obtusifolia* samples with different scaling methods; Figure S4: Loadings plot of PC2 vs. PC1 with assignment of all 24 aminosteroids described in our previous study [2]. Figure S5–S35: Spectral data of aminosteroids identified in this study.

## Author Statement

This work is part of the doctoral thesis of Justus W. Mukavi.

## Abbreviations

The following abbreviations are used in this manuscript:

*B. obtusifolia*	*Buxus obtusifolia*
UHPLC–ESI^+^–QqTOF–MS/MS	Ultra-high-performance liquid chromatography coupled with positive-mode electrospray ionization double quadrupole time-of-flight tandem mass spectrometry
LC/MS	Shortform for UHPLC–ESI^+^–QqTOF–MS/MS
QC	Quality control
*t*_R_	Retention time
Min	Minutes
MVDA	Multivariate data analysis
PCA	Principal component analysis
PC	Principal component
ABA	Abscisic acid
