# Supplementary material for: Seasonal and Organ-Specific Variations of Alkaloids in Buxus obtusifolia (Mildbr.) Hutch: A Multivariate LC/MS Study
Source: Plants (Basel). 2026 May 8;15(10):1439. doi: 10.3390/plants15101439 (PMC13211022; doi:10.3390/plants15101439)
Supplement: Supplementary file 1 [file plants-15-01439-s001.zip › plants-4279436-supplementary.pdf]

plants

MDPI

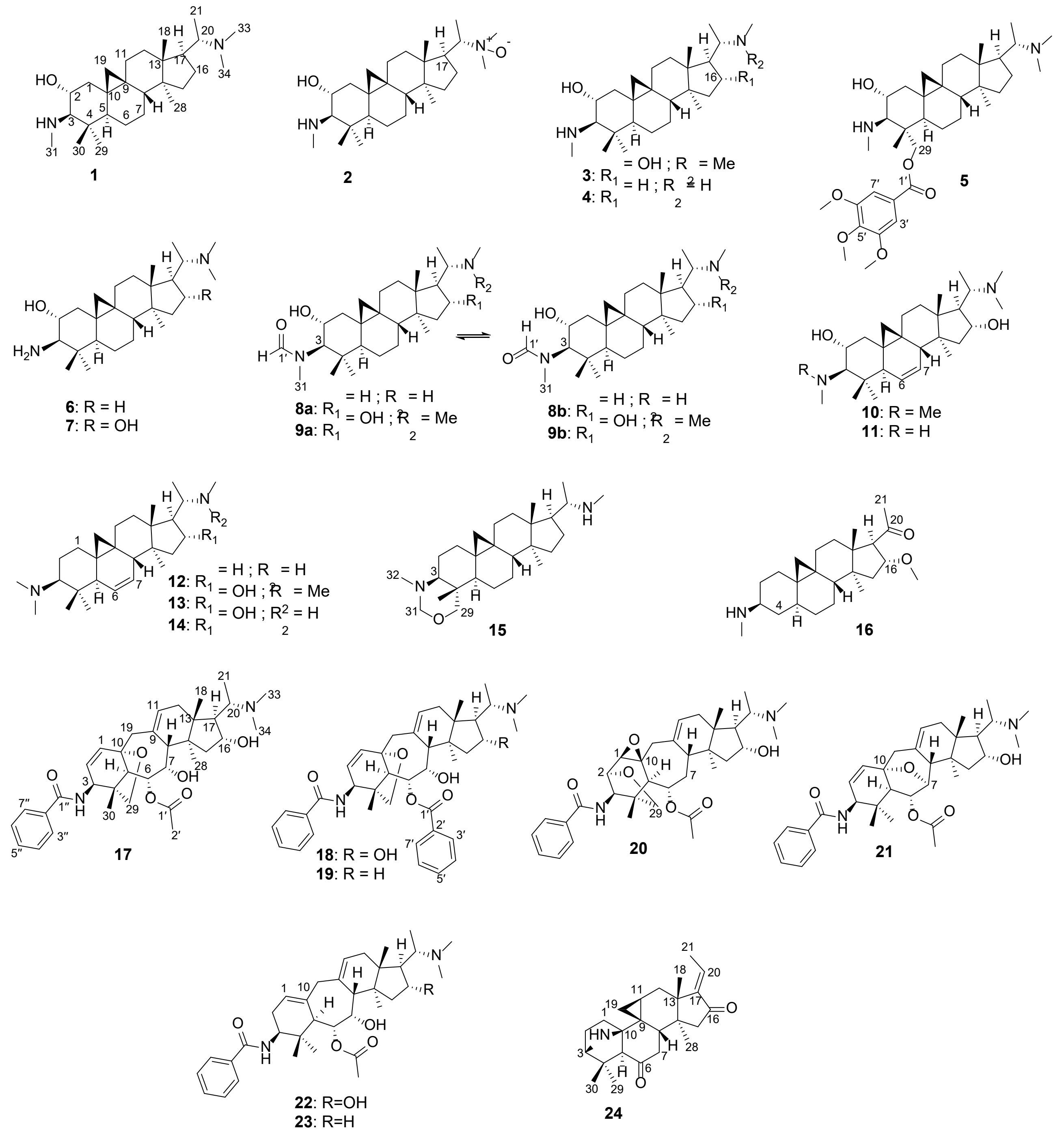

Figure S1. Chemical structures of aminosteroids previously isolated from *Buxus obtusifolia* in our recent study [2].

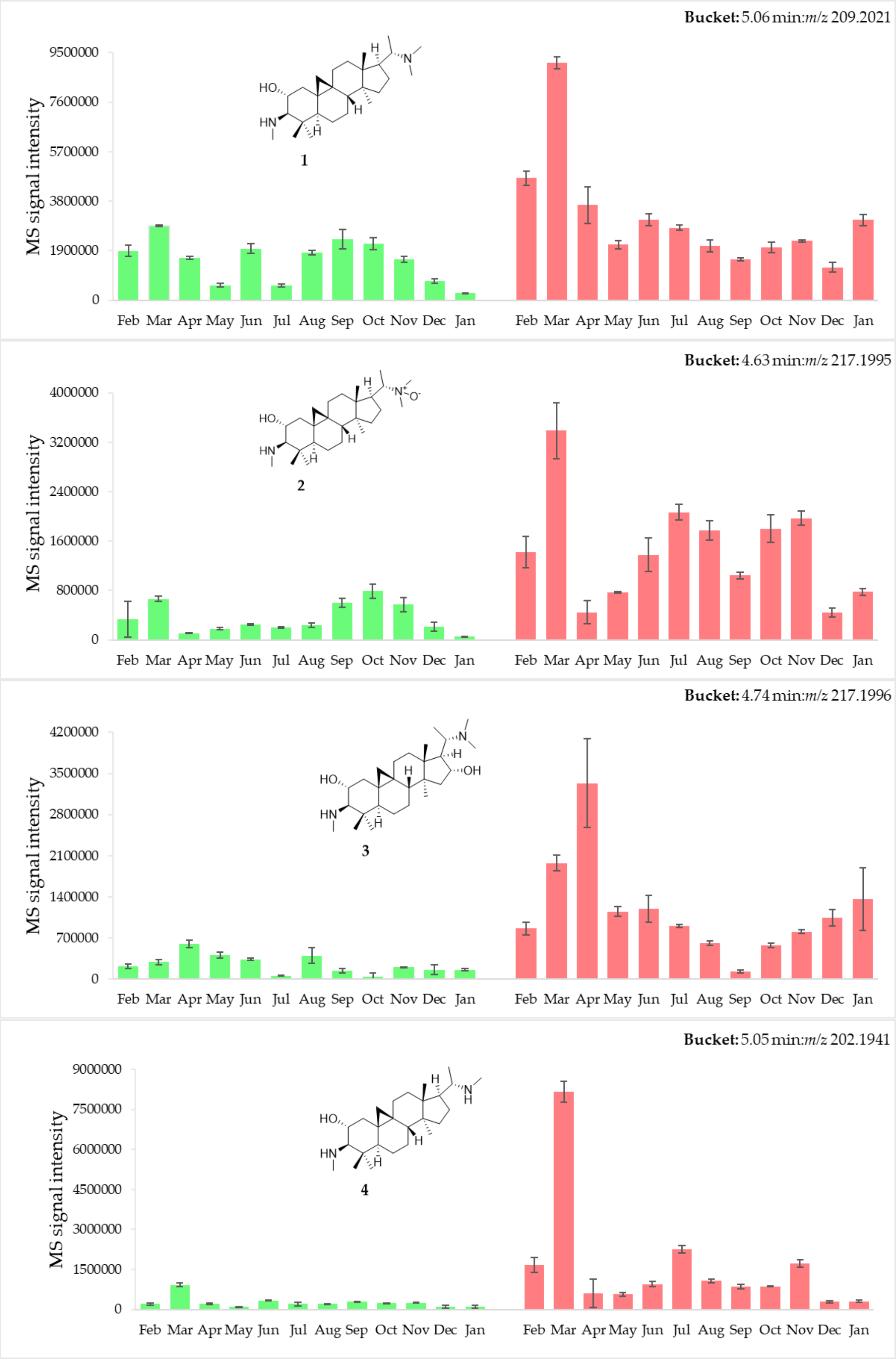

Figure S2. Continued.

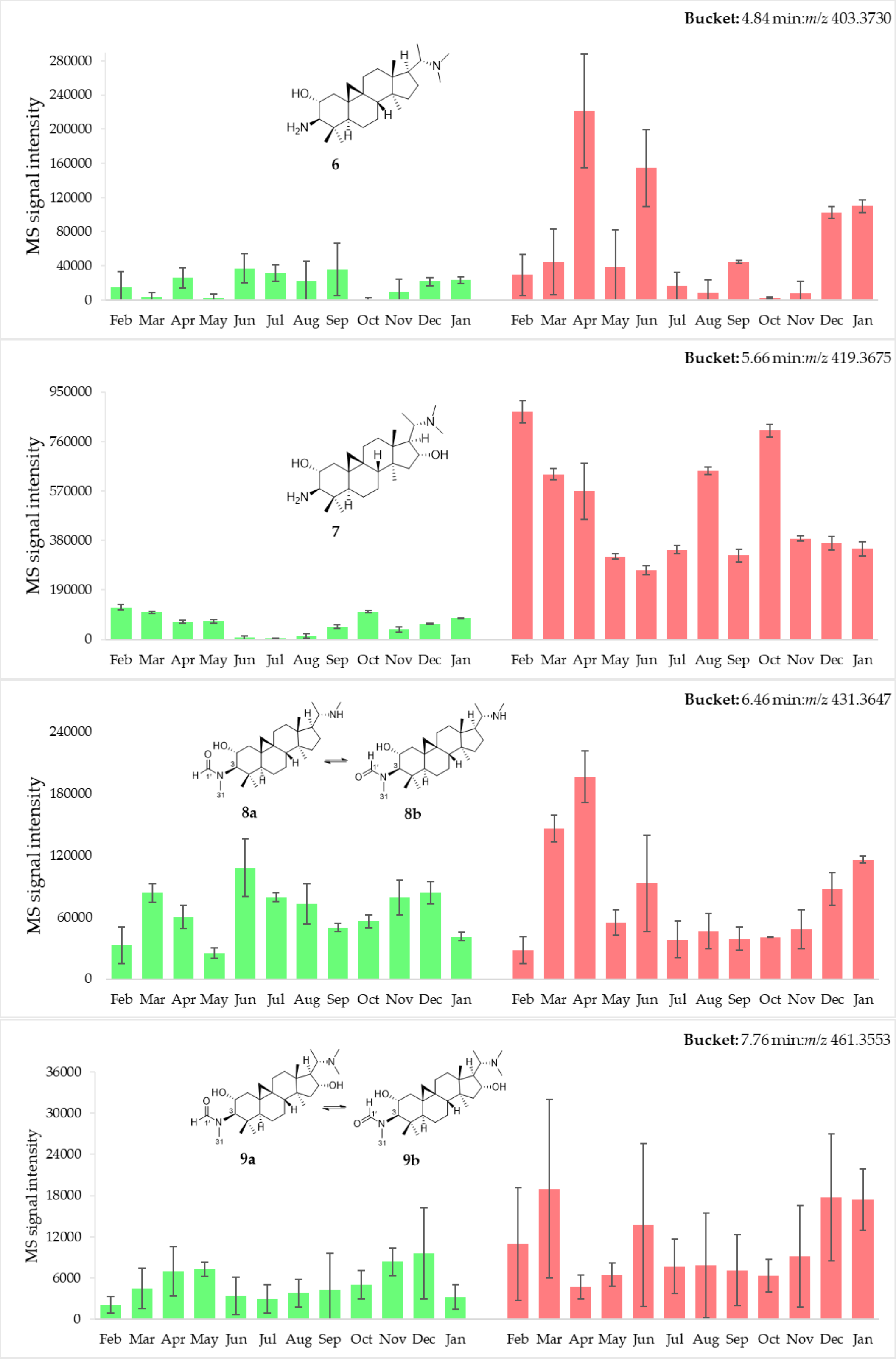

Figure S2. Continued.

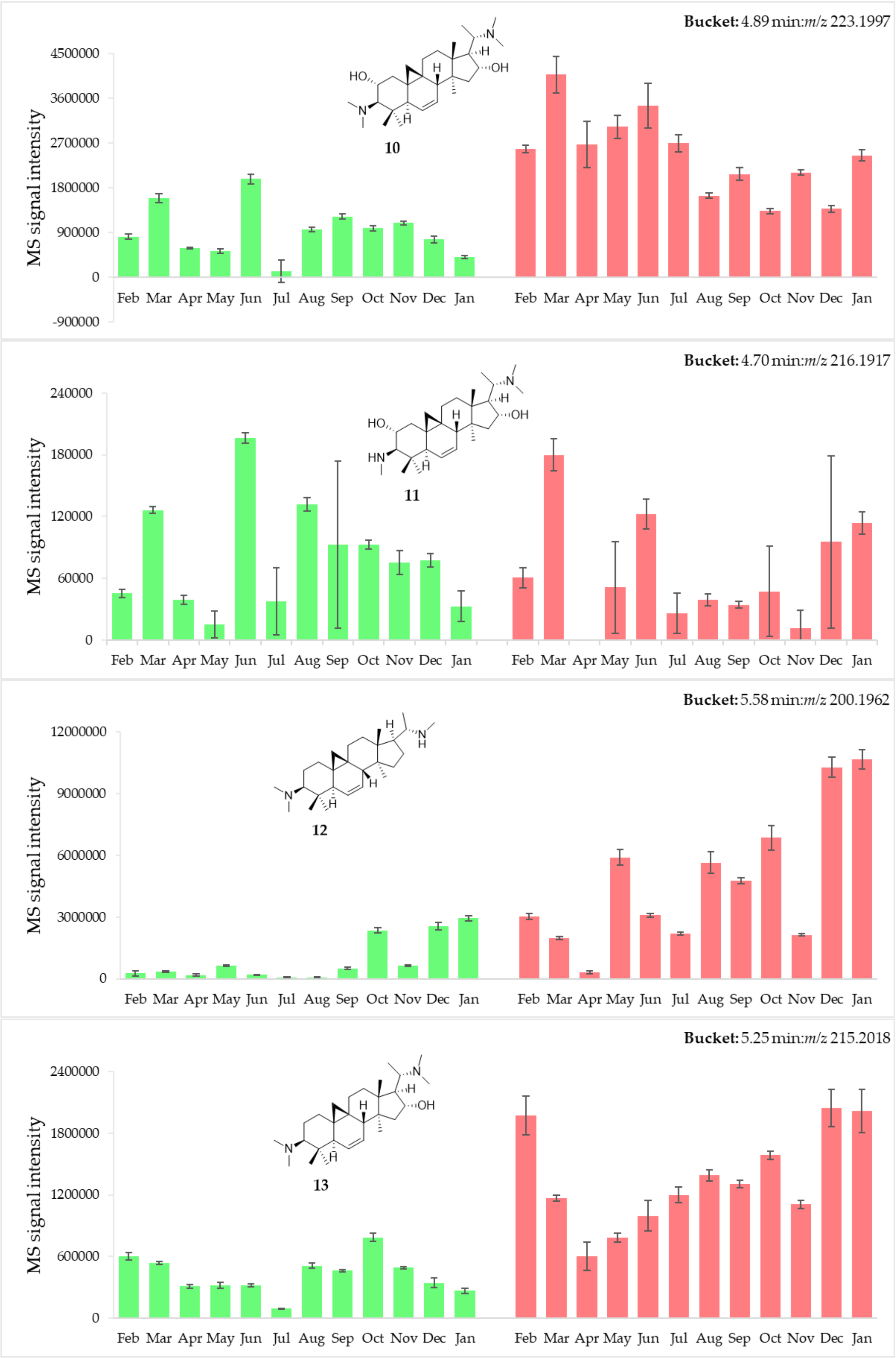

Figure S2. Continued.

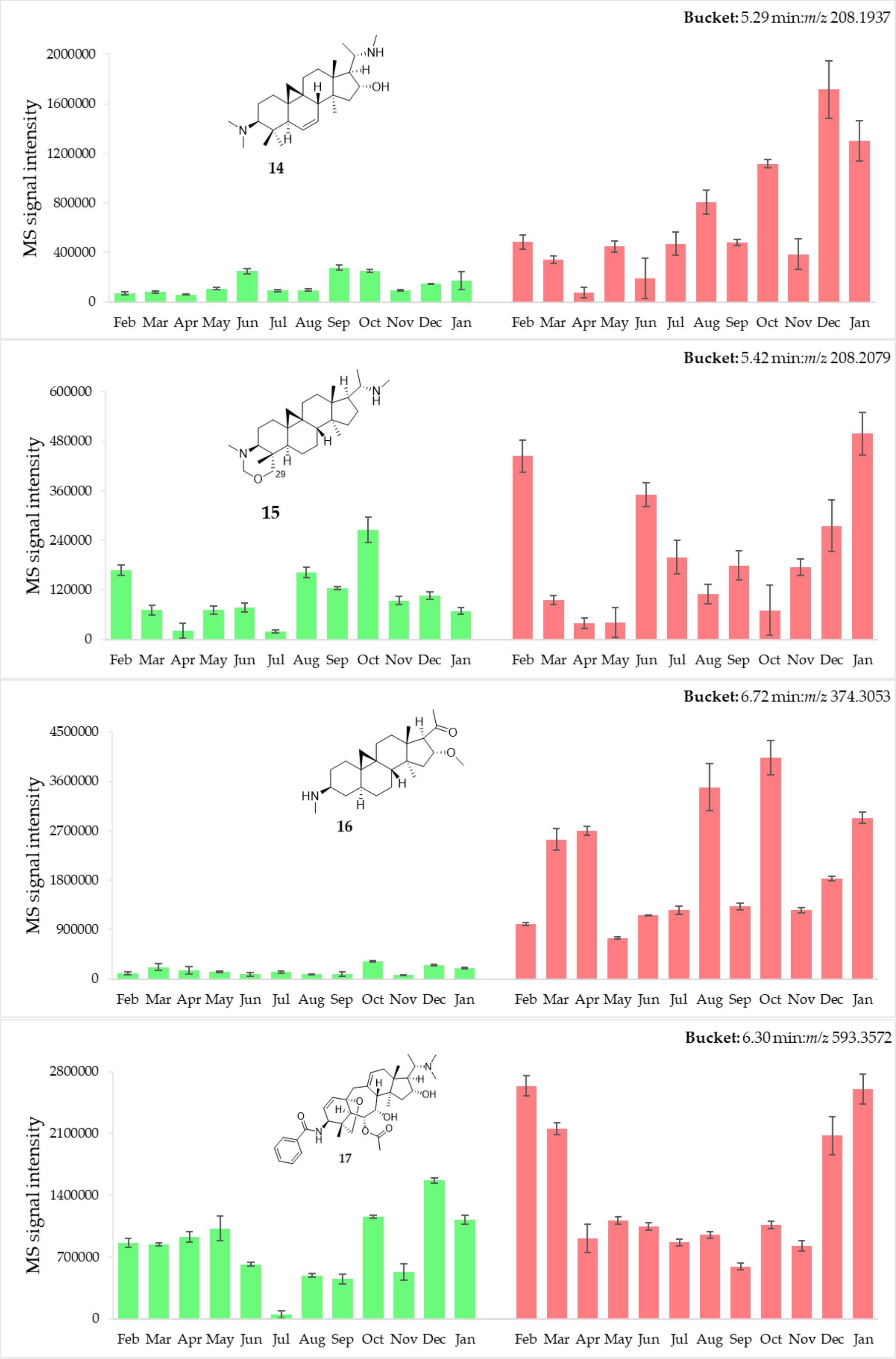

Figure S2. Continued.

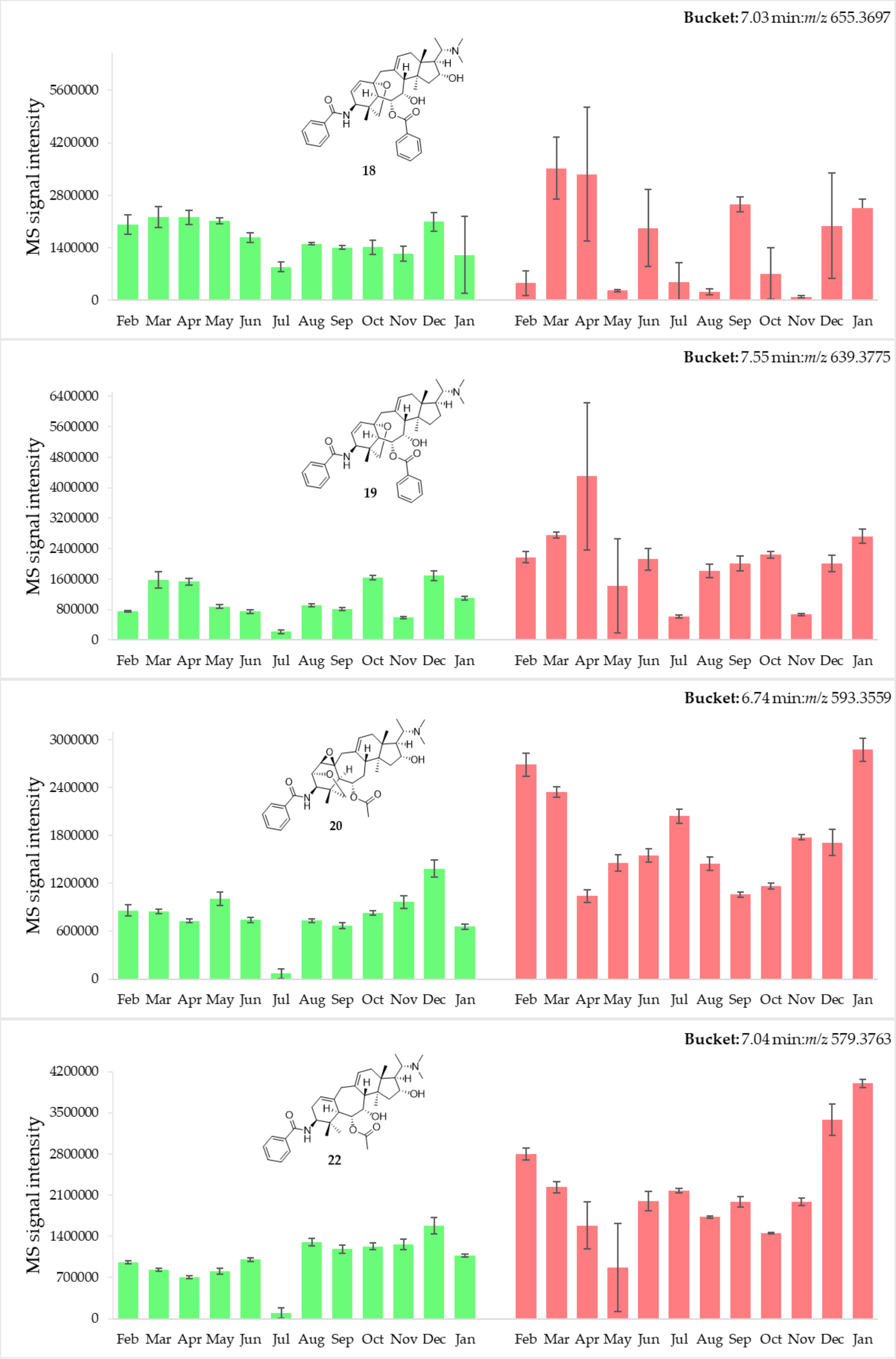

Figure S2. Continued.

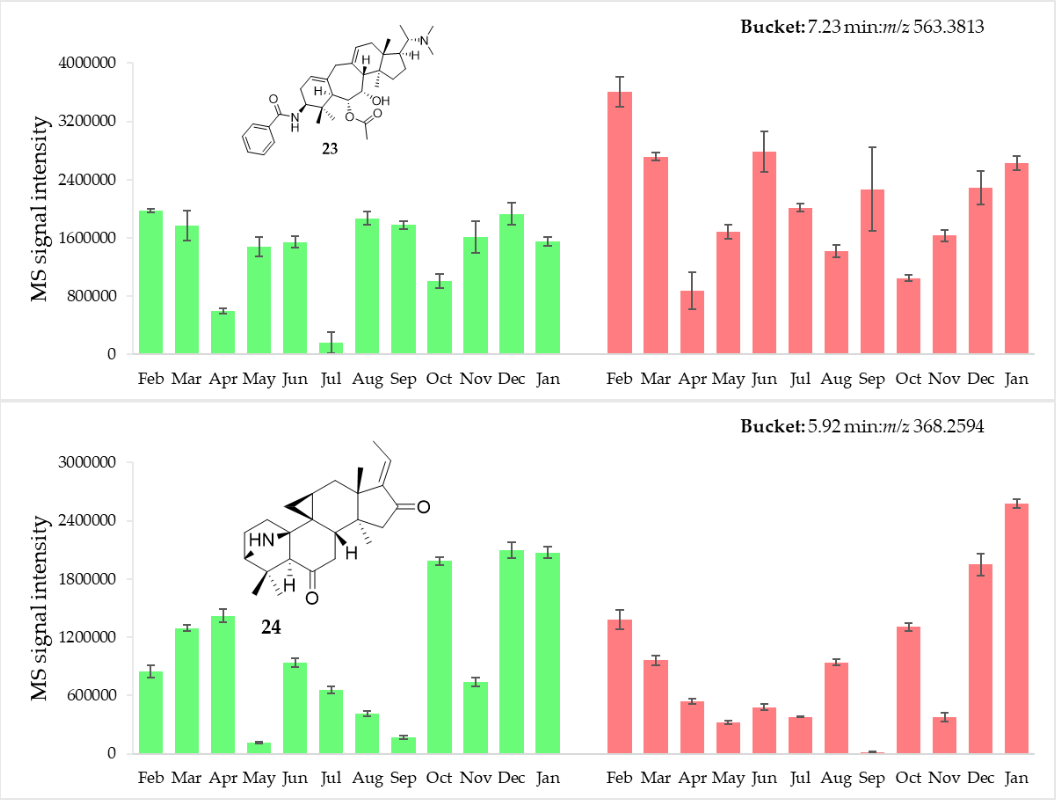

Figure S2. Monthly profiles of compounds previously isolated from *B. obtusifolia* [2]. Bar charts are the mean signal intensities (± SD) of three replicate measurements across twelve monthly samples (February 2024–January 2025) of *B. obtusifolia* twigs (green) and leaves (red).

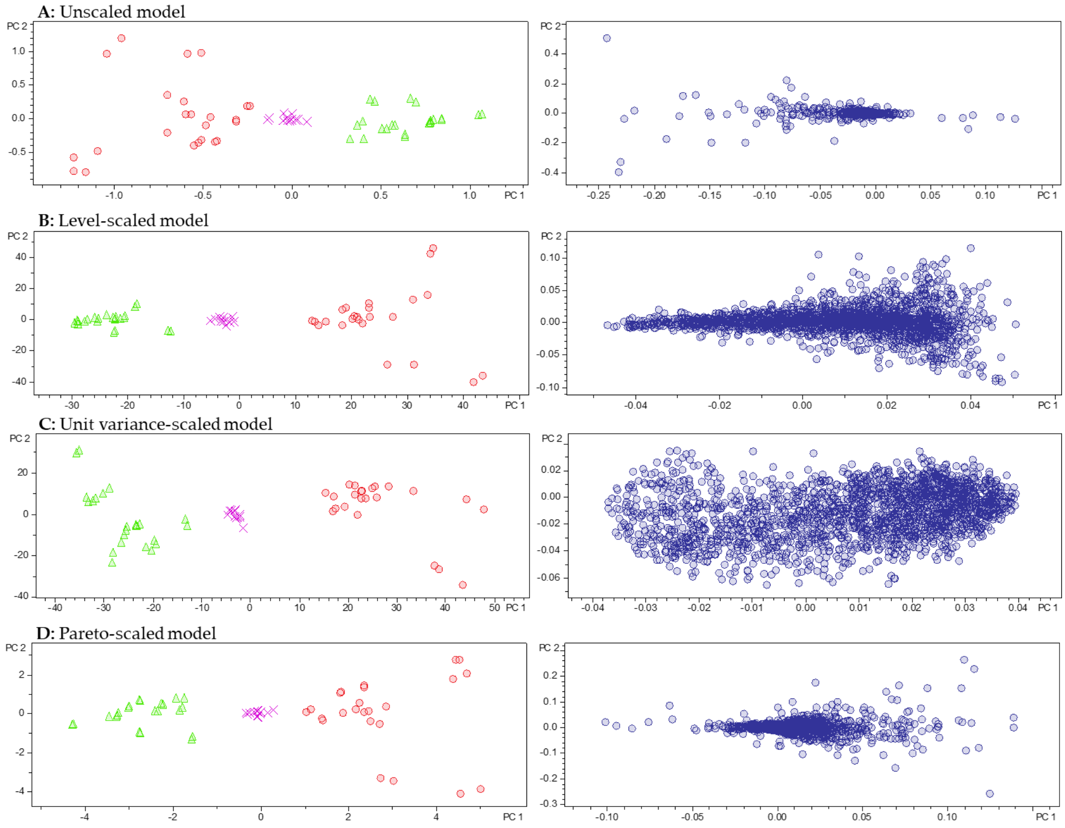

Figure S3. PCA models of the LC/MS data of *B. obtusifolia* samples with different scaling methods (A–D). Scores plot left, loadings plot right. Legend: red = leaves; green = twigs, pink = quality control mix.

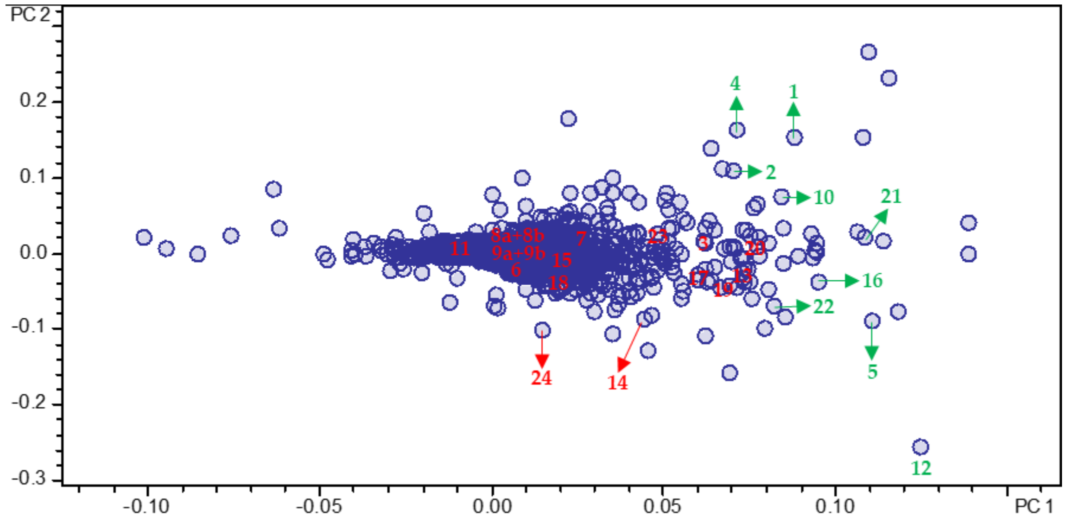

Figure S4. Loadings plot of the pareto-scaled PCA model, PC2 vs. PC1 with assignment of the buckets representing the 24 aminosteroids described in our previous study [2], compare Figure S1. Several of these compounds were found to exhibit particularly high variance and were therefore highlighted in green.

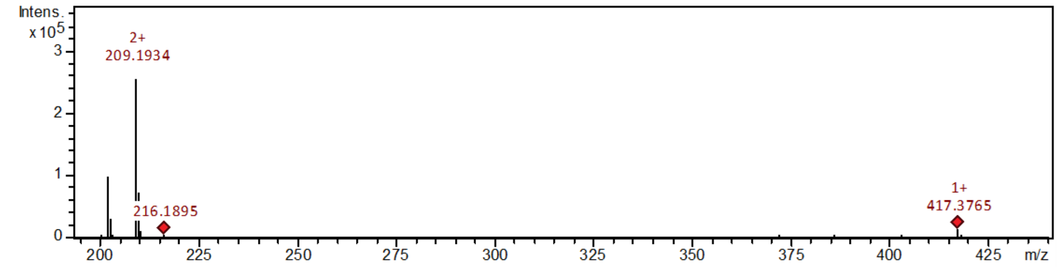

Figure S5. +ESI-QqTOF MS spectrum of compound **1** (cycloprotobuxoline-C [2]); *m/z* 209.1934 $[M+2H]^{2+}$, 417.3765 $[M+H]^+$.

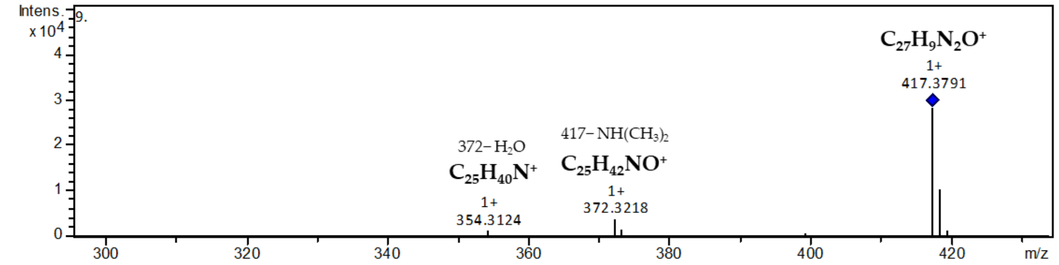

Figure S6. +ESI-QqTOF MS/MS spectrum of the $[M+H]^+$ ion of compound **1** (cycloprotobuxoline-C [2]).

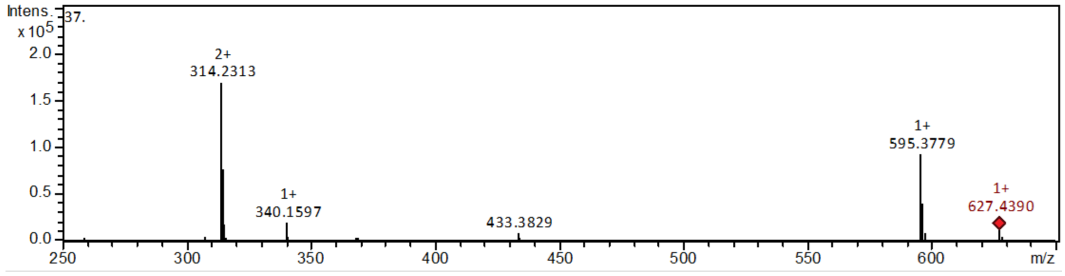

Figure S7. +ESI-QqTOF MS spectrum of compound **5** (29-trimethoxybenzoyloxy cycloprotobuxoline-C [2]); *m/z* 314.2313 $[M+2H]^{2+}$, 627.4390 $[M+H]^+$.

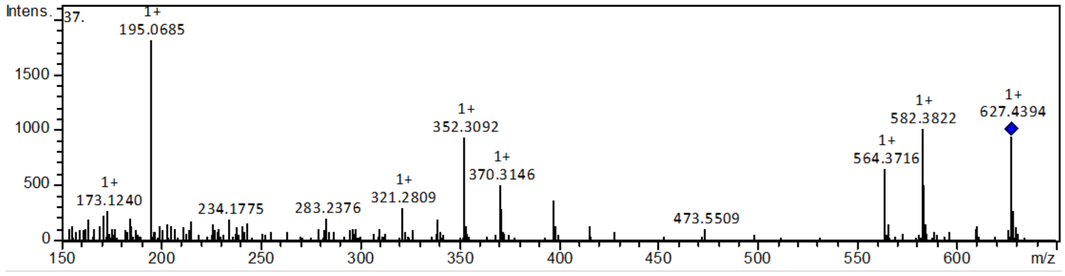

Figure S8. +ESI-QqTOF MS/MS spectrum of the $[M+H]^+$ ion of compound **5** (29-trimethoxybenzoyloxy cycloprotobuxoline-C [2]).

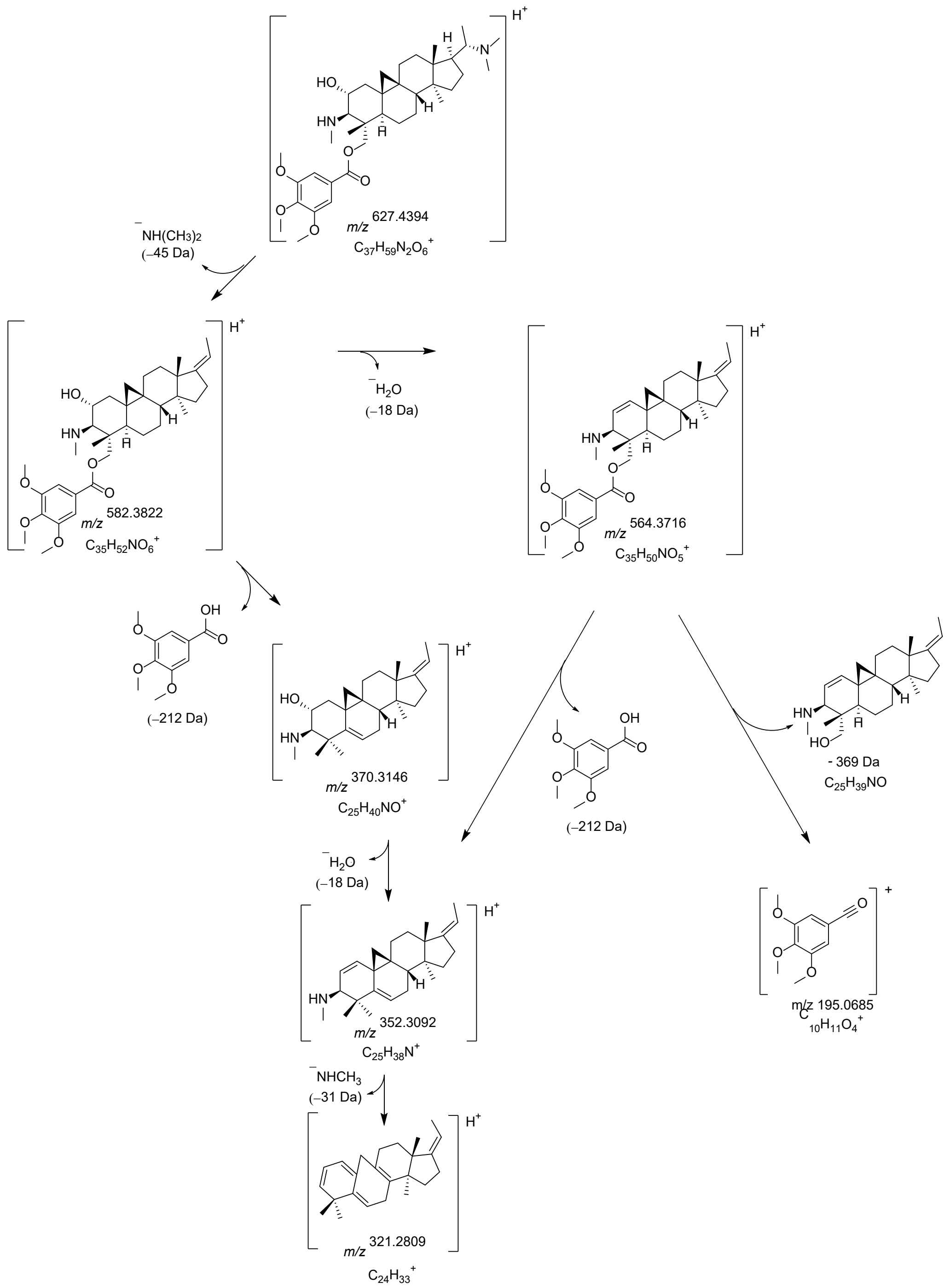

Figure S9. Postulated fragmentation pathway of the [M+H]⁺ ion of compound **5** (29-trimethoxybenzoyloxy cycloprotobuxoline C).

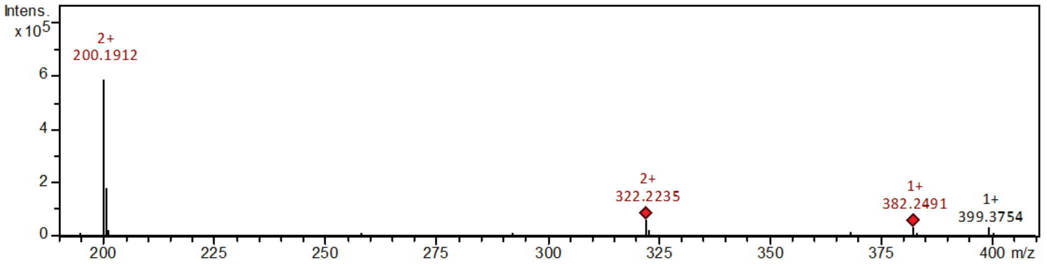

Figure S10. +ESI-QqTOF MS spectrum of compound **12** (deoxycyclovirobuxeine-B [2]); *m/z* 200.1912 $[M+2H]^{2+}$, 399.54 $[M+H]^{+}$.

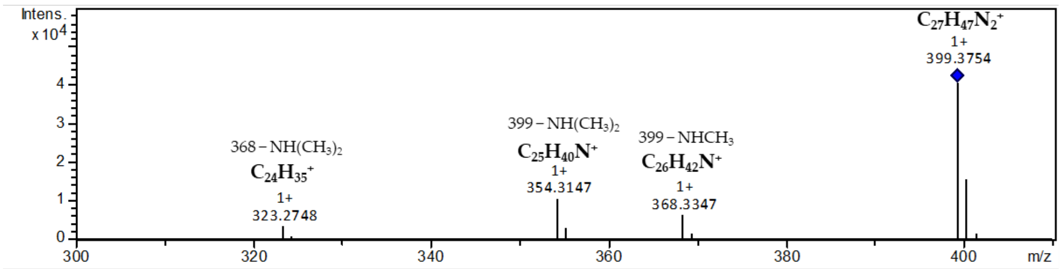

Figure S11. +ESI-QqTOF MS/MS spectrum of the $[M+H]^+$ ion of compound **12** (deoxycyclovirobuxeine-B [2]).

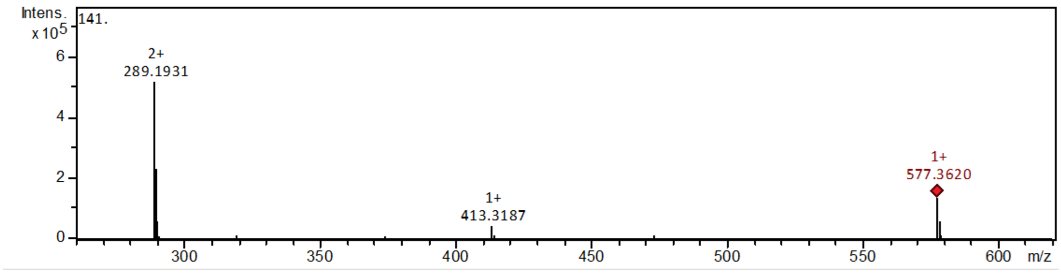

Figure S12. +ESI-QqTOF MS spectrum of compound **21** (obtusiepoxamine-A [2]); *m/z* 289.1931 $[M+2H]^{2+}$, 577.3620 $[M+H]^+$.

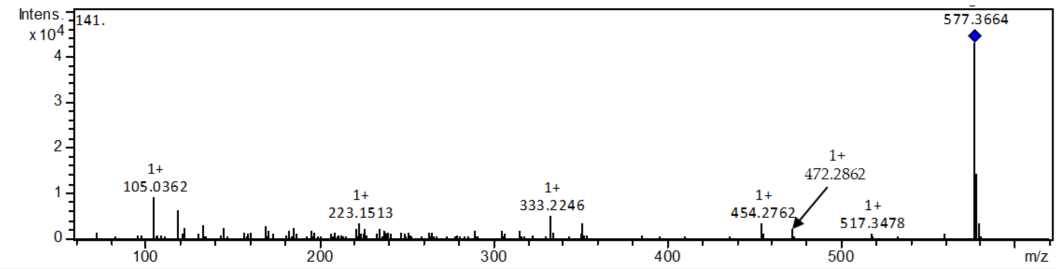

Figure S13. +ESI-QqTOF MS/MS spectrum of the $[M+H]^+$ ion of compound **21** (obtusiepoxamine-A [2]).

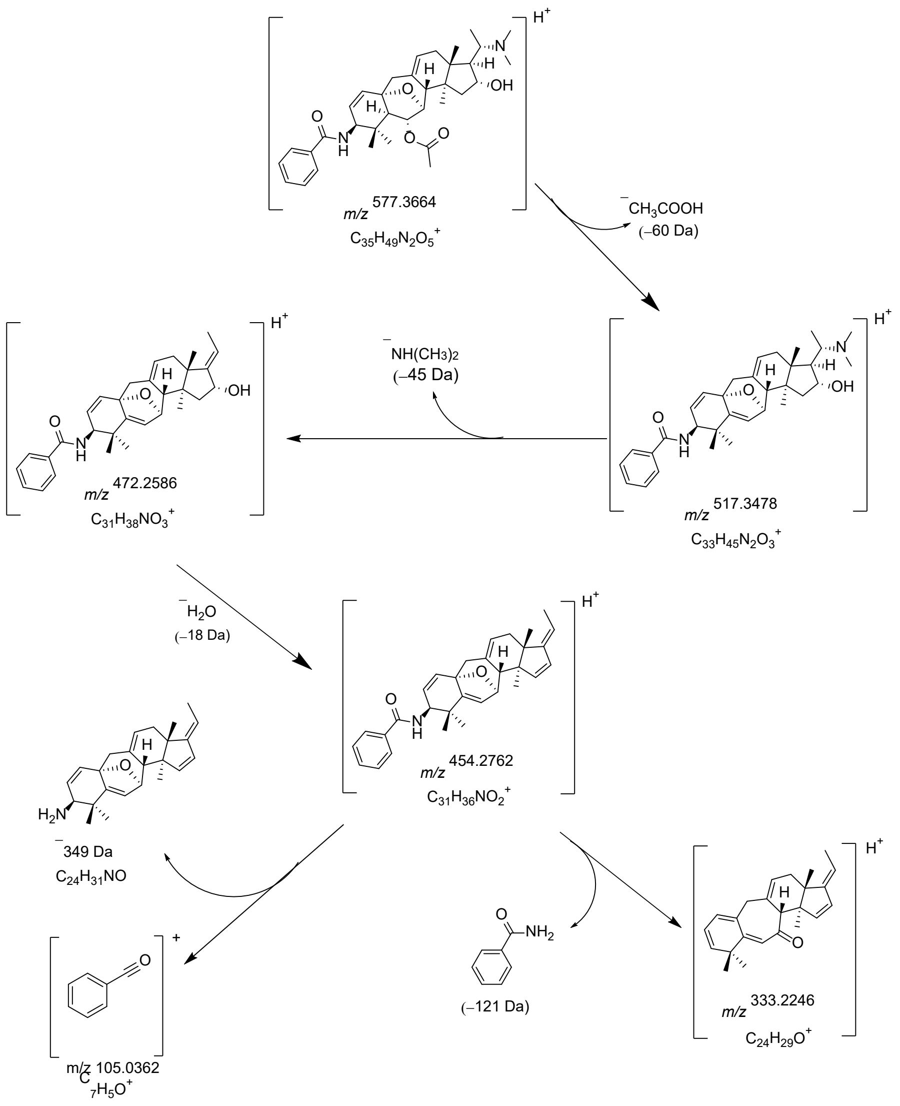

Figure S14. Postulated fragmentation pathway of the $[M+H]^+$ ion of compound **21** (obtusiepox-amine-A).

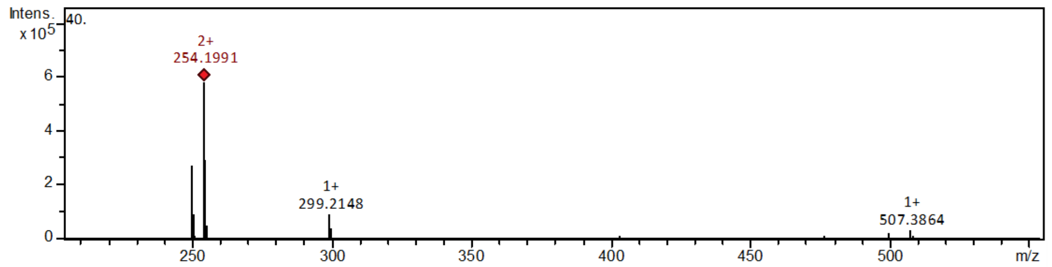

Figure S15. +ESI-QqTOF MS spectrum of *O*-benzoyl-cycloprotobuxoline-D.

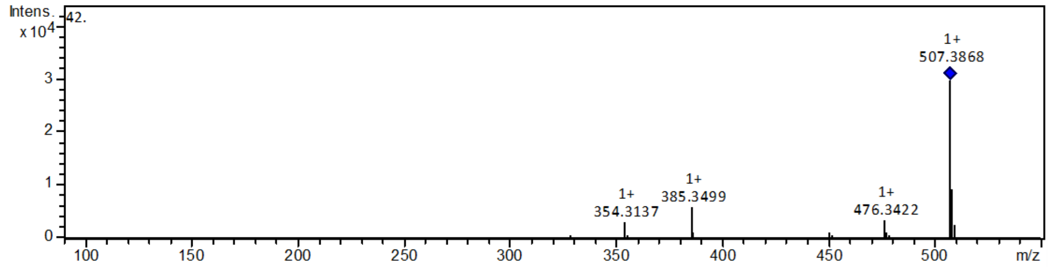

Figure S16. +ESI-QqTOF MS/MS spectrum of the $[M+H]^+$ ion of *O*-benzoyl-cycloprotobuxoline-D.

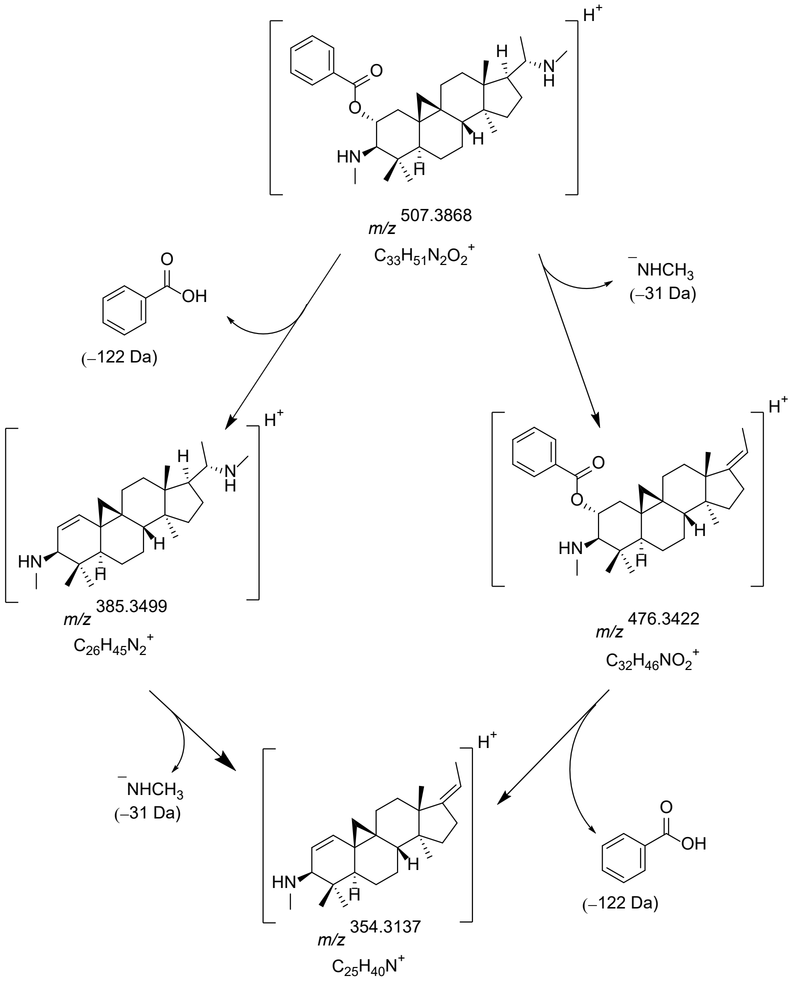

Figure S17. Postulated fragmentation pathway of the [M+H]⁺ ion of *O*-benzoyl-cycloprotobuxoline-D.

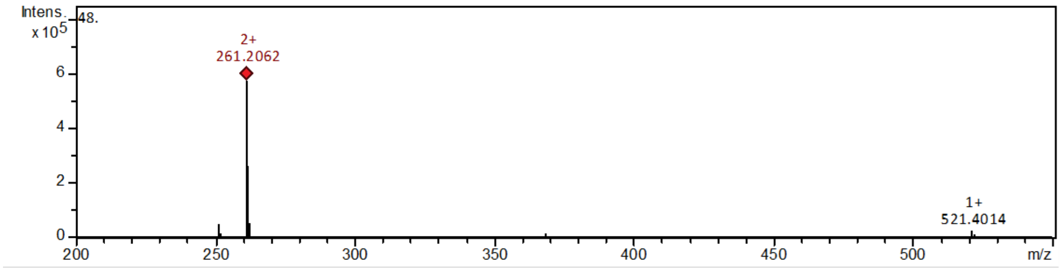

Figure S18. +ESI-QqTOF MS spectrum of *N*-benzoyl-cycloprotobuxolin-C.

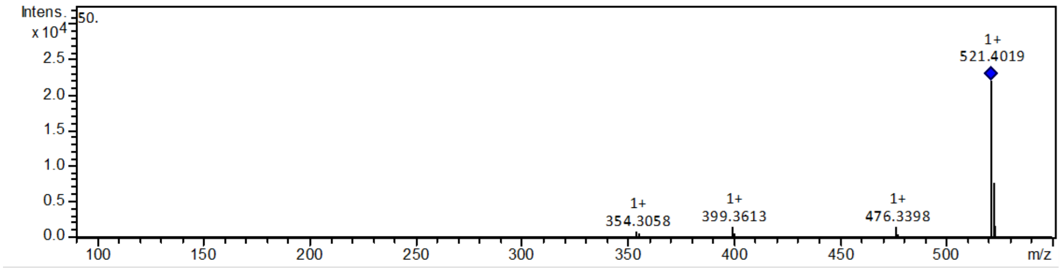

Figure S19. +ESI-QqTOF MS/MS spectrum of the [M+H]⁺ ion of *N*-benzoyl-cycloprotobuxolin-C.

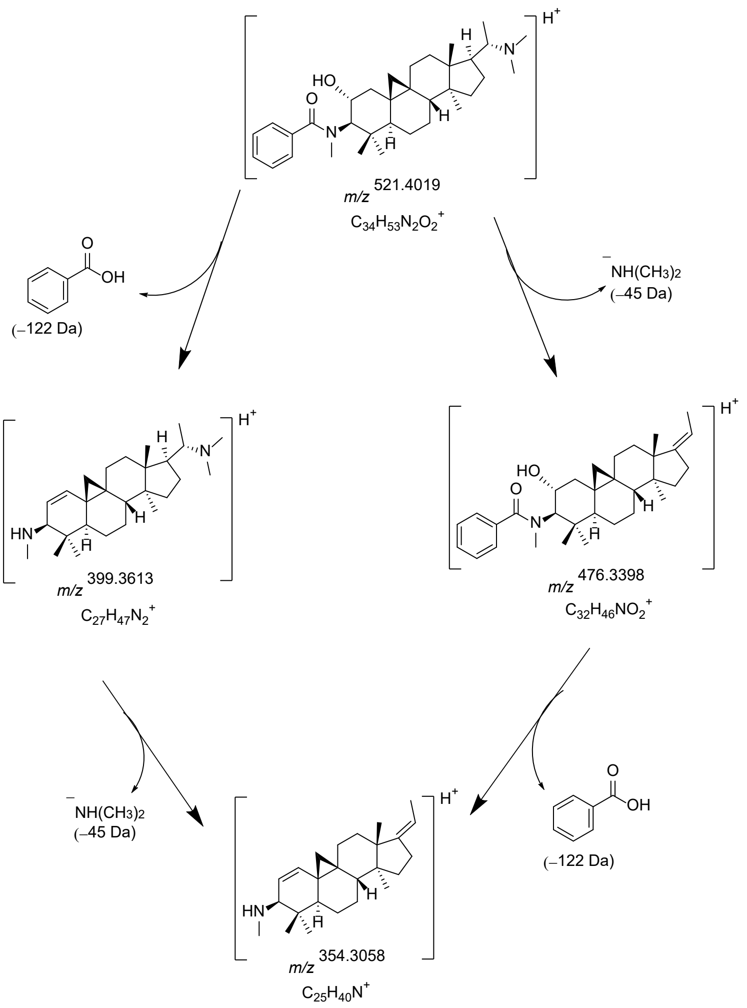

Figure S20. Postulated fragmentation pathway of the $[M+H]^+$ ion of *N*-benzoyl-cycloprotobuxolin-C.

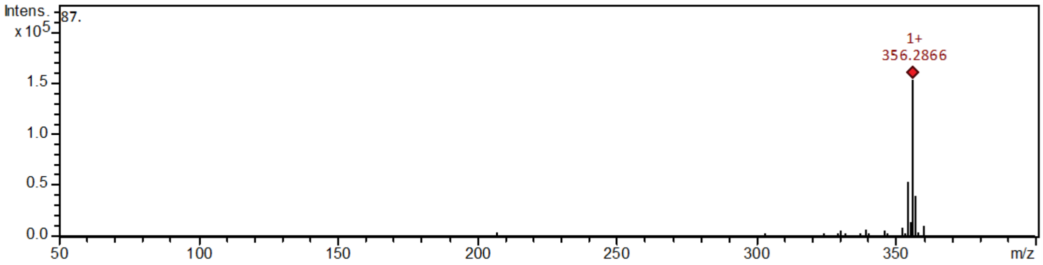

Figure S21. +ESI-QqTOF MS spectrum of cyclobuxophylline O.

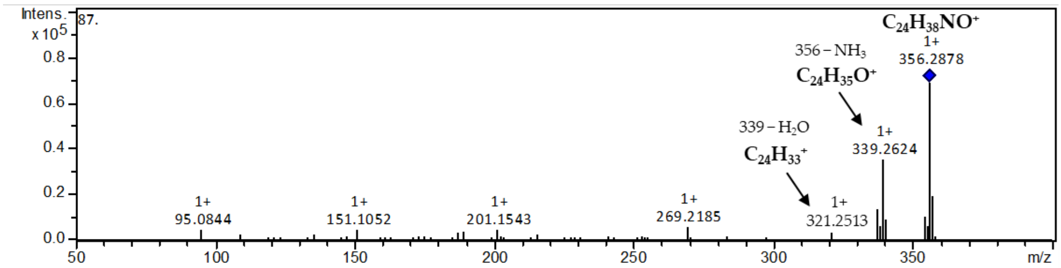

Figure S22. +ESI-QqTOF MS/MS spectrum of the $[M+H]^+$ ion of cyclobuxophylline O.

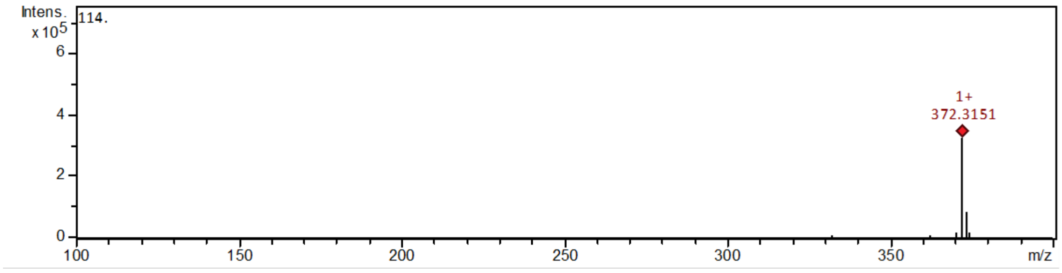

Figure S23. +ESI-QqTOF MS spectrum spectrum of buxtauine M.

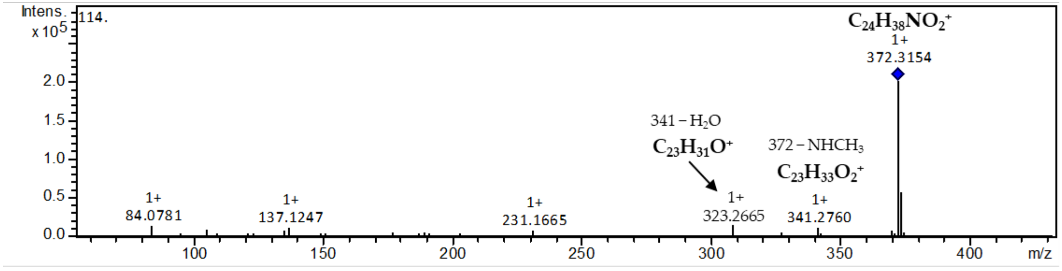

Figure S24. +ESI-QqTOF MS/MS spectrum of the [M+H]$^+$ ion of buxtauine M.

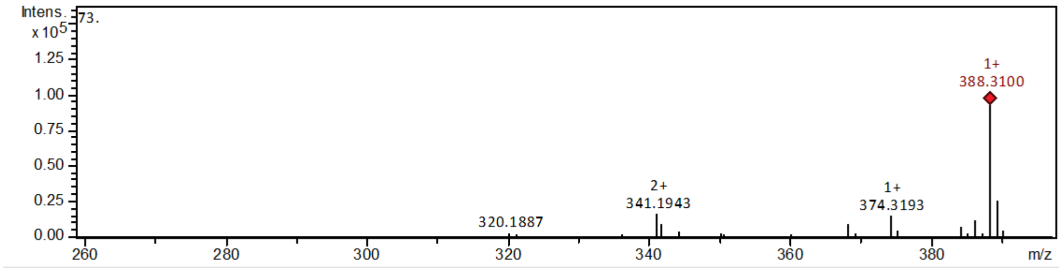

Figure S25. +ESI-QqTOF MS spectrum of buxaustroine A.

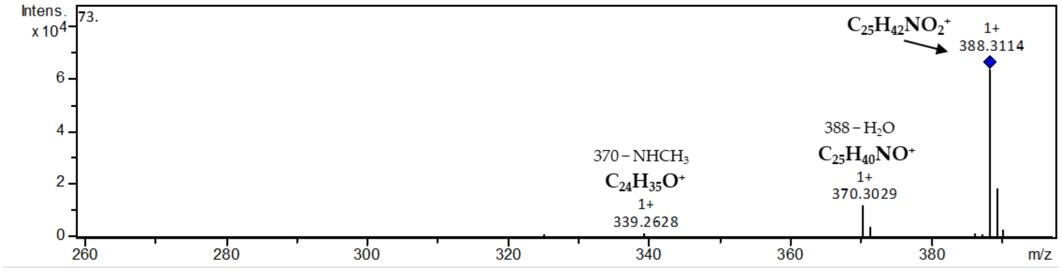

Figure S26. +ESI-QqTOF MS/MS spectrum of the [M+H]$^+$ ion of buxaustroine A.

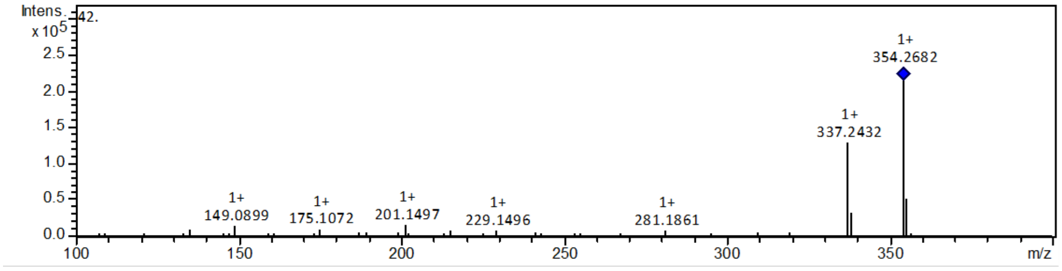

Figure S27. +ESI-QqTOF MS/MS spectrum of the [M+H]$^+$ ion of bucket: 6.63 min:*m/z* 354.2682

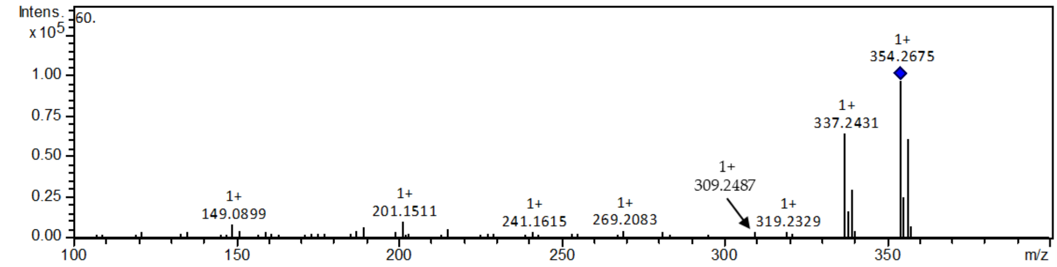

Figure S28. +ESI-QqTOF MS/MS spectrum of the $[M+H]^+$ ion of bucket: 6.92 min:*m/z* 354.2675

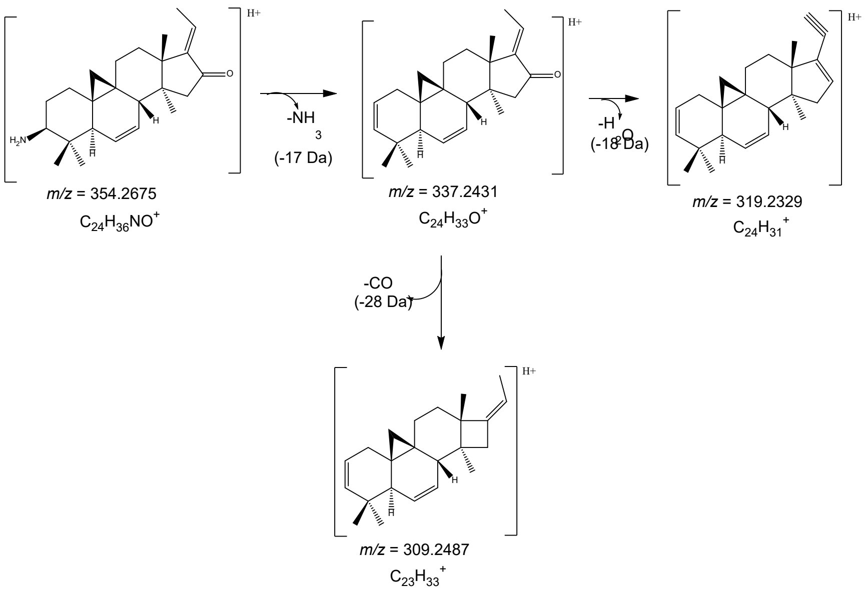

Figure S29. Postulated fragmentation pathway of the $[M+H]^+$ ion of bucket: 6.92 min:*m/z* 354.2675.

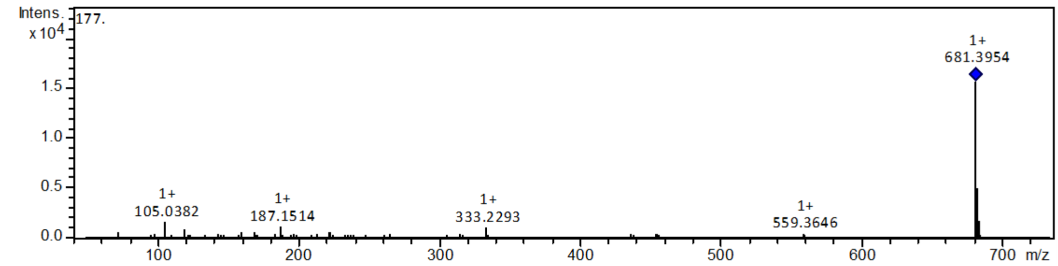

Figure S30. +ESI-QqTOF MS/MS spectrum of the $[M+H]^+$ ion of bucket: 8.00 min:*m/z* 681.3954

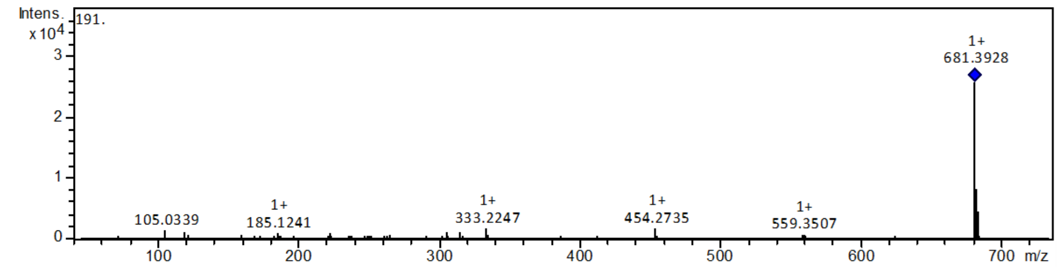

Figure S31. +ESI-QqTOF MS/MS spectrum of the $[M+H]^+$ ion of bucket: 8.27 min:*m/z* 681.3928

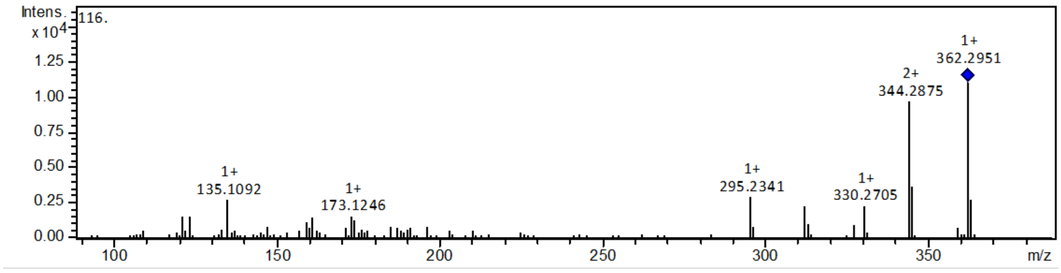

Figure S32. +ESI-QqTOF MS/MS spectrum of the $[M+H]^+$ ion of bucket: 7.68 min:*m/z* 362.2951

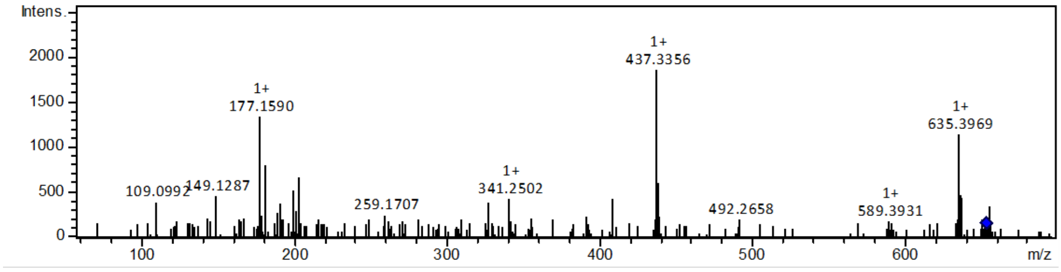

Figure S33. +ESI-QqTOF MS/MS spectrum of the $[M+H]^+$ ion of bucket: 10.86 min:*m/z* 653.4137

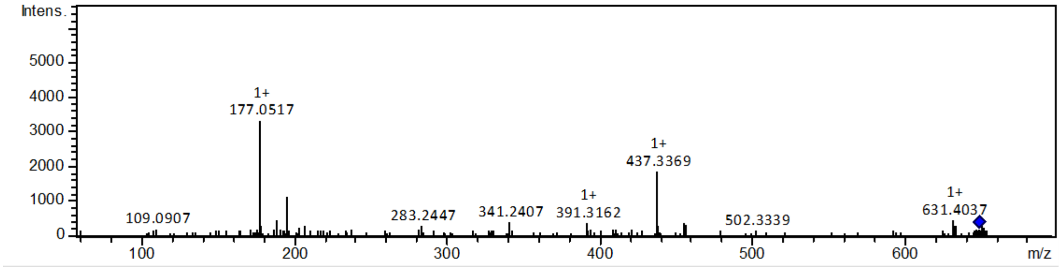

Figure S34. +ESI-QqTOF MS/MS spectrum of the $[M+H]^+$ ion of bucket: 11.12 min:*m/z* 649.4200

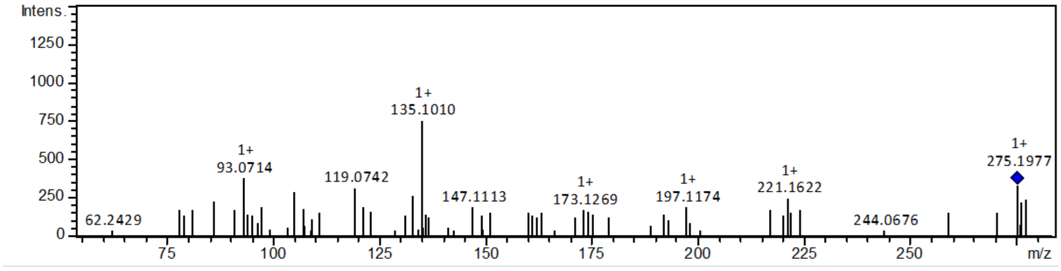

Figure S35. +ESI-QqTOF MS/MS spectrum of the $[M+H]^+$ ion of bucket: 9.77 min:*m/z* 275.1977
